# Effect of membrane performance variability with temperature and feed composition on pervaporation and vapor permeation system design for solvent drying

**DOI:** 10.1002/jctb.7161

**Published:** 2022-06-15

**Authors:** Leland M Vane

**Affiliations:** U.S. Environmental Protection Agency, Office of Research and Development, Center for Environmental Solutions and Emergency Response, Cincinnati, OH, USA

**Keywords:** solvent reclamation, membrane permeability, membrane processes, dehydration, pervaporation, vapor permeation, membrane-based separation

## Abstract

**BACKGROUND:**

The presence of water in organic solvents and biofuels can complicate their production and reuse because many hydrophilic solvents form difficult-to-separate mixtures with water (e.g., azeotropes). Pervaporation (PV) and vapor permeation (V⋅P) remove water from such mixtures via selective solution-diffusion transport through a membrane material. A recent article reviewed design factors that impact the effectiveness of PV/V⋅P solvent dehydration processes (*J. Chem. Technol. Biotechnol* 95: 495–512 (2020)). For the sake of simplicity, the earlier work assumed constant membrane permeabilities. The objective here is to explore the impact of variable permeabilities on predictions of PV/V⋅P system performance.

**RESULTS:**

A multiparameter expression relating permeability to process conditions was incorporated into the spreadsheet calculators from the previous work. Use of the expression was demonstrated with literature ethanol/water PV data for a NaA zeolite material and two poly (vinyl alcohol) (PVA) membranes. The variable permeabilities of the membranes yielded membrane area requirements that were 20–30% different from those calculated using permeances fixed at either end of the target water range. The impact of composition-dependent permeabilities was most pronounced on the fraction of ethanol transferred to the permeate for the NaA membrane.

**CONCLUSION:**

The inclusion of membrane permeabilities that vary with fluid composition and temperature noticeably altered predictions of the membrane area required to carry out water removal from ethanol by PV and of the transfer of ethanol to the permeate stream. Unless a PV/V⋅P process is expected to operate at a constant temperature and in a narrow concentration range, process performance estimates would be improved by inclusion of concentration- and temperature-dependent permeabilities or permeances.

## INTRODUCTION

**Figure 1.**
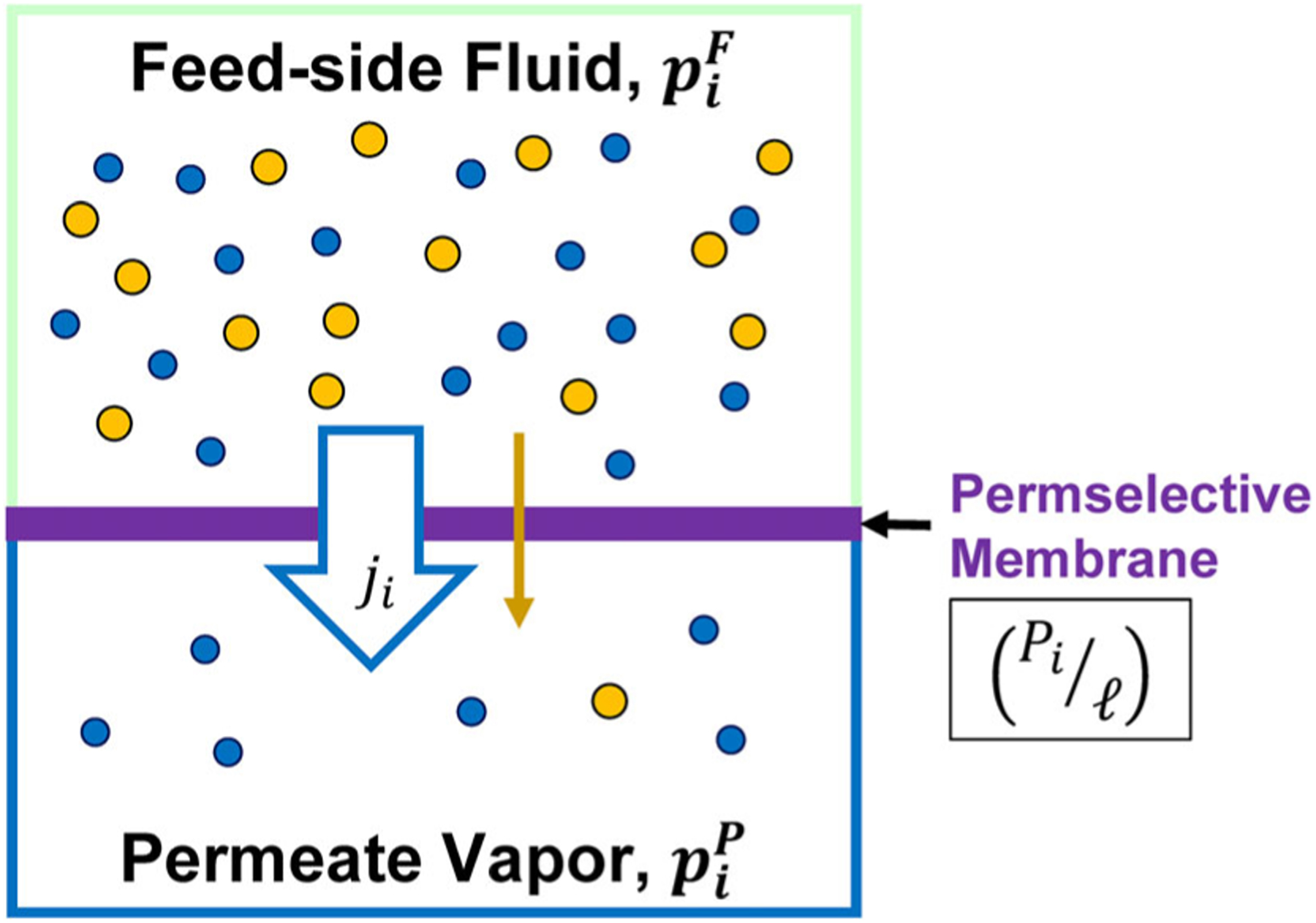
Illustration of mass transfer in PV and V⋅P processes with flux through permselective membrane. The molar flux of a compound through the membrane $\left(j_{i}\right)$ is dictated by the ratio of the permeability $\left(P_{i}\right)$ to the thickness (ℓ) of the selective layer (i.e., ‘permeance’) multiplied by the difference in the partial pressure of the compound from the feed side to the permeate side of the membrane $\left(p_{i}^{F}-p_{i}^{P}\right)$.

**Figure 2.**
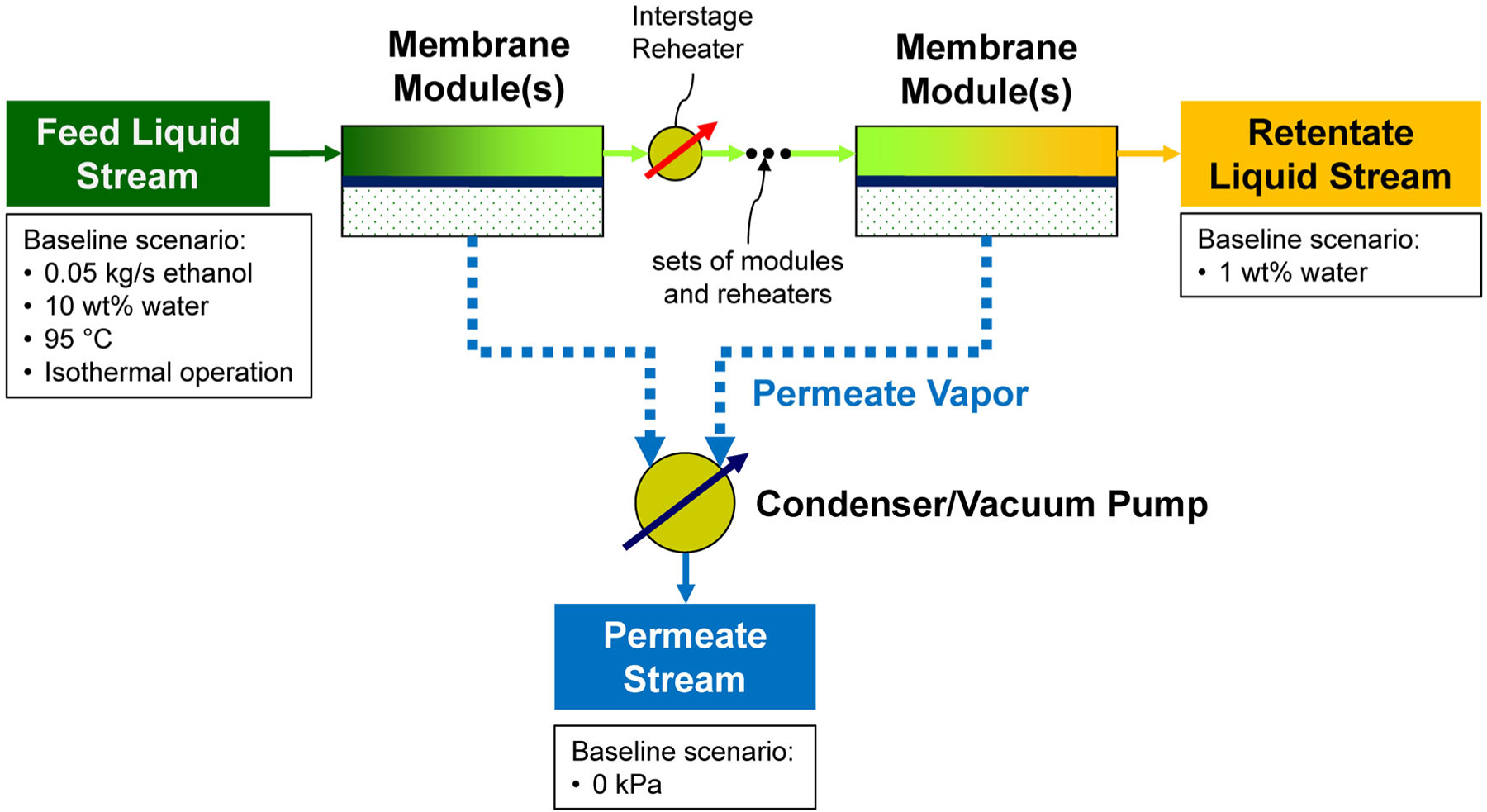
Illustration of single-pass PV solvent drying process. Assumptions for the baseline ethanol drying scenario are shown.

Common industrial organic solvents and biofuels, such as ethanol, 2-propanol, and 1-butanol, form difficult-to-separate mixtures with water (including azeotropic mixtures), making their production and reclamation challenging. In most of these mixtures, water is the minor component. For example, the atmospheric pressure ethanol-water azeotrope contains 4.0 wt% water. The nascent membrane processes of pervaporation (PV) and vapor permeation (V⋅P) can efficiently remove water from these difficult-to-separate mixtures by relying on selective permeation transport through dense hydrophilic membrane materials combined with a process design that provides the driving force for the permeation transport. The transport of compounds from the feed-side fluid through the PV/V⋅P membrane into the permeate vapor is illustrated in Fig. 1. Two recent articles by this author reviewed permselective membrane materials available for PV/V⋅P solvent dehydration systems and the factors that impact the design and effectiveness of an overall process based on such membranes.^1,2^ A continuous, single-pass PV process is illustrated in Fig. 2, showing a feed fluid entering a multi-module membrane system that produces a water-depleted retentate stream containing the solvent to be reused and a water-enriched permeate stream. Process factors included the properties of the membrane, properties of the solvent/water mixture, and operating parameters. Detailed spreadsheet calculators were developed for single-pass and recirculating batch PV systems and for single-pass V⋅P systems to provide estimates of membrane area, permeate concentrations, solvent recovery, permeate condenser temperatures, and heating requirements. The spreadsheet calculators associated with the previous review article are available as Supporting Information for that article^2^ (updated spreadsheet calculators are available as Supporting Information for the present article). Although a recirculating batch V⋅P process is technically possible, as noted in the previous process factor article, such a system is not particularly practical due to the large amount of energy required to evaporate and condense the recirculating stream through the multiple passes needed to reach the desired water level. Process variables included: solvent type, feed flow rate (single-pass process) or initial amount of material (batch process), water permeance, solvent permeance (or water/solvent selectivity), initial and final water concentrations in the feed-side fluid, feed temperature (for PV) or feed pressure (for V⋅P), temperature drop due to evaporation (for PV) or feed-side pressure drop (for V⋅P) and permeate pressure. From the analyses in the process factor paper, it was concluded that PV/V⋅P process performance is dependent on a wide range of process parameters and that a change in one process condition can result in changes to all process outcomes, particularly when non-ideal conditions, such as non-zero permeate pressure, are involved.^2^

For simplicity, the spreadsheet calculators in the earlier work assumed constant membrane permeabilities for the solvent and water. This assumption made the calculations and analyses more straightforward. However, permeabilities are known to change to some degree with fluid composition and temperature. Changes in permeability with water concentration variations are most commonly associated with hydrophilic polymers, like poly(vinyl alcohol) (PVA),^3–9^ but inorganic membranes consisting of zeolite and hybrid silica selective layers also exhibit performance changes with composition and temperature.^10–15^ In most materials, the compounds in the feed fluid interact with the membrane in a way that alters the transport properties of the membrane. For example, a compound might absorb into a polymer to an extent that causes swelling of the polymer and that swelling thus results in increased permeabilities for all compounds.^16–18^ For inorganic membranes, matrix swelling is greatly reduced relative to polymers, but phenomena, such as competitive adsorption, slowed transport, and alteration of effective interstitial pore diameter, can impact transport through the material.^10,12,19–23^ For PV processes, temperature plays a major role in determining the driving force for transport through the membrane due to the impact of temperature on the vapor pressure of the compounds. However, even when membrane performance has been corrected for driving force and reported in terms of permeability or permeance, temperature variability in membrane performance is still observed.^6,9,11,13,24,25^ As a result, in V⋅P processes, where feed-side partial pressure is the most important parameter in determining transport driving force, temperature is still a relevant factor.

The objective of this work is to expand upon the previous process factor effort by exploring the impact of variable permeabilities on PV/V⋅P system design and performance. This will involve establishing an adaptable expression relating permeability to process conditions, incorporating the expression into the PV/V⋅P spreadsheet calculators from the previous work, using literature PV data to illustrate the use of the permeability expression, and then assessing how variable permeances impact the predicted system performance as estimated with the process calculators.

## MEMBRANE TRANSPORT MODEL

The transport of compounds from the bulk feed-side fluid through a PV or V⋅P membrane is illustrated in Fig. 1 and represented with the following relationship:

(1) $$j_{i}=\frac{P_{i}}{\ell }\left(p_{i}^{F}-p_{i}^{P}\right)$$

where $j_{i}$ is the molar flux (kmol m^−2^ s^−1^) of compound i through the membrane. The membrane performance characteristic is $P_{i}$, the permeability of compound i through the membrane selective layer (kmol m m^−2^ s^−1^ kPa^−1^). The thickness of the selective layer of the membrane is ℓ(m). The thickness is often provided in terms of μm since that is the order of magnitude of most selective layers. The ratio $P_{i}/\ell$ is the permeance of the membrane, $\Pi_{i}$. SI units for permeance are kmol m^−2^ s^−1^ kPa^−1^ but the common unit is the Gas Permeation Unit (GPU), where 1 kmol m^−2^s^−1^ kPa^−1^ = 2.99 × 10^9^ GPU. Herein, permeance will be reported in units of GPU.

The driving force for transport through the membrane is the difference in the partial pressure, in kPa, of the compound on the feed-side of the membrane, $p_{i}^{F}$, and on the permeate-side of the membrane, $p_{i}^{p}$. The permeate-side partial pressure is simply calculated as the product of the permeate mole fraction $\left(y_{i}\right)$ and total permeate pressure, $p_{\text{Total }}^{p}$ (*i.e*., $p_{i}^{p}=y_{i}p_{\text{Total }}^{p}$). All pressures are in terms of absolute pressure. Mass flux, $J_{i}$, in units of kg m^−2^ h^−1^, is a commonly reported performance metric for PV and V⋅P membranes. It is related to molar flux as follows:

(2) $$J_{i}=M_{i}j_{i}\left(3600\text{ s}/\text{h}\right)$$

where $M_{i}$ is the molecular weight(kg kmol^−1^) of compound i. The molar permselectivity of the membrane (hereafter, ‘selectivity’) for one compound relative to another, $\alpha_{ij}$, is defined as the ratio of permeabilities or, equivalently, the permeances of the two compounds:

(3) $$\alpha_{ij}=\frac{P_{i}}{P_{j}}=\frac{\Pi_{i}}{\Pi_{j}}$$

### Permeability expression

As noted above, in the previous review article on the effect of process factors on the solvent dehydration performance of PV and V⋅P systems, membrane permeability was assumed to be constant for a given membrane/solvent combination. This assumption simplified the calculations and made it more straightforward to highlight the effect of other process factors, such as temperature, water concentration, permeate pressure, and solvent type, on system design. The assumption of constant permeability was also influenced by the wide variety of empirical and theory-based expressions relating the permeability of membrane materials to process conditions proposed in the literature.^8,17,26–39^ Many of these expressions were specific to the membrane or membrane/species combination of the study. The PV and V⋅P dehydration process spreadsheet calculators developed by the author were intended for use with a variety of solvents and membranes. The calculators require a valid set of pure component physical property parameters (heat capacity, enthalpy of vaporization, vapor pressure), water-solvent vapor-liquid equilibrium thermodynamic parameters, and membrane performance information (solvent and water permeances). Due to this variety of solvents and membranes, a flexible expression for permeability was required. After examining the aforementioned expressions from the literature, and with an emphasis on adaptability, the following expression relating permeability to the feed-side activities of the compounds and the feed-side temperature was integrated into the process calculator spreadsheets:

(4) $$P_{i}=P_{oi}\text{exp}\left[\frac{E_{i}}{R}\left(\frac{1}{T_{o}}-\frac{1}{T}\right)\right](A_{i}+B_{i}a_{w}+C_{i}a_{w}{\;}^{2}+D_{i}a_{s}+F_{i}a_{s}{\;}^{2})\text{exp}\left(G_{i}a_{w}+H_{i}a_{s}\right)$$

Composition is represented in this expression by the activities of the solvent and water in the feed fluid, $a_{s}$ and $a_{w}$, respectively, defined here as the ratio of the partial pressure of a compound under feed-side conditions to the saturated vapor pressure of that compound at the same temperature. The expression for $\Pi_{i}$ is simply Eqn (4) divided by the actual, or assumed, membrane thickness. Eqn (4) allows for a variety of relationships between permeability and the following activities: constant, linear, quadratic, or exponential. The parameters $P_{oi},A_{i},B_{i},C_{i},D_{i},E_{i},F_{i},G_{i}$, and $H_{i}$ are constants for compound i with a specific membrane and establish the form of the relationship being used. The relationship between permeability and feed-side temperature T(K) in Eqn (4) is an Arrhenius-type term with exponential parameter $E_{i}(\;kJ\;\left.{kmol}^{-1}\right)$, reference temperature $T_{o}(\;K)$, and gas constant $R\;\left(8.314\;kJ{kmol}^{-1}{\;K}^{-1}\right)$.

Activity was selected as the composition-based parameter primarily because it is commonly used in the modeling of vapor sorption, diffusion, and/or permeability in polymeric and microporous inorganic materials.^16,17,31,40–45^ In addition, unlike vapor pressure, activity is nearly independent of temperature for a fixed solvent/water composition in PV,^46^ allowing the Arrhenius-type term to represent temperature variability. The activity in the feed-side vapor of a V.P process and the activity in the feed-side liquid of a PV process are calculated differently.

In V⋅P, the feed-side partial pressure is calculated as follows:

(5) $$p_{i}^{F}=x_{i}p_{\text{Total}}^{F}$$

where $x_{i}$ is the mole fraction in the feed fluid, $p_{\text{Total }}^{F}$ is the total feed-side pressure, and ideal gas behavior is assumed (i.e., the fugacity coefficient = 1). As noted earlier, activity is the ratio of $p_{i}^{F}$ to the saturated vapor pressure of that compound $\left(p_{i}^{\text{sat }}\right)$. Thus, feed-side activity for V⋅P is as follows:

(6) $$a_{i}\left(\text{ V}\cdot \text{P}\right)=\frac{x_{i}p_{\text{Total }}^{F}}{p_{i}^{\text{sat }}}$$

In PV, the feed-side partial pressure is the partial pressure of a compound in a hypothetical vapor that is in equilibrium with the feed-side liquid $\left(p_{i}^{F^{*}}\right)$, defined as follows:

(7) $$p_{i}^{F}=p_{i}^{F^{\text{*}}}=x_{i}\gamma_{i}p_{i}^{\text{sat }}$$

where $\gamma_{i}$ is the activity coefficient of compound i in the feed-side liquid. Thus, unlike V⋅P, the feed-side partial pressures in a PV system are not a function of the applied feed-side pressure. Instead, they are a function of temperature (via $p_{i}^{\text{sat }}$), composition, and the interaction between the solvent and water. The activity of a compound in the PV feed-side liquid is then:

(8) $$a_{i}\left(\text{PV}\right)=x_{i}\gamma_{i}$$

In this work, the Non-Random Two-Liquid (NRTL) thermodynamic model will be used to calculate $\gamma_{i}$ for binary solvent-water mixtures and the Antoine equation will be used to calculate $p_{i}^{\text{sat }}$. NRTL and Antoine parameters for the solvents of interest in this work are provided in the Supporting Information (Tables S1 and S2). Parameters for calculating liquid heat capacity and enthalpy of vaporization are listed in Tables S3 and S4. Alternative sources for these parameters are provided below Tables S1–S4.

Given this information about partial pressures, Eqn (1) can be rewritten for V⋅P and PV operations as follows:

(9) $$\text{Vapor Permeation }:j_{i}=\frac{P_{i}}{\ell }\left(x_{i}p^{F}-y_{i}p^{P}\right)$$

(10) $$\text{Pervaporation }:j_{i}=\frac{P_{i}}{\ell }\left(x_{i}\gamma_{i}p_{i}^{\text{sat }}-y_{i}p^{P}\right)$$

The hope is that some form of Eqn (4) will portray the composition- and temperature-dependence observed in data for a solvent-water-membrane system of interest. Parameters in Eqn (4) are determined by fitting a form of the equation to experimental data that covers the range of conditions anticipated for the solvent drying process and has been converted to membrane permeabilities. For any given situation, many, or even most, of the parameters will be zero. Only the least complicated form of the equation that adequately represents the performance for the desired purpose should be used. The ultimate simplification would be a membrane with constant permeability — independent of temperature (i.e., $E_{i}$ is zero) and feed fluid composition (i.e., $B_{i},C_{i},D_{i},F_{i},G_{i}$, and $H_{i}$ are all zero). Although unusual, the latter has been reported for some amorphous perfluorinated polymers.^8,38^ Ignoring temperature or composition effects on permeability might also be appropriate for systems with small changes in those properties.

The most common simplification to Eqn (4) for solvent dehydration systems is likely the assumption of no dependence on solvent activity (i.e., $D_{i},F_{i}$, and $H_{i}$ in Eqn (4) would be zero). It is expected that permeability dependence on the activity of water will be adequate to capture variations with feed composition for most solvent dehydration systems. The solvent parameters were included in Eqn (4) in case there is some dependence on solvent activity. For PV systems with binary mixtures, solvent and water activities cannot be independently varied since changing the composition of one component in a binary liquid mixture automatically changes that of the other component. As a result, it might be impossible to differentiate dependence on solvent activity from dependence on water activity from PV data sets. For V⋅P systems, the two activities can be independently varied, even for binary mixtures, through a combination of composition, total pressure, and temperature changes. Thus, V⋅P testing is more amenable to separation of the dependence of permeability on water and solvent activities. For example, ethanol/water V⋅P separation performance of a water-selective UV-treated polybutadiene membrane developed in the author’s group was observed to depend on the activities of both water and ethanol.^47^

As indicated in Eqns (9) and (10), calculations of PV or V⋅P system performance require knowledge of the permeability and membrane thickness or the ratio of the two (i.e., permeance) to calculate the fluxes of water and solvent through the membrane. For many membranes, the exact thickness of the selective layer is unknown and only permeance values are applicable. In those cases, a hypothetical thickness can be assumed to calculate working permeability values from permeance values and vice versa. This default thickness value must be carried over into process calculations so that the original permeance values are recovered. In this work, a hypothetical thickness of 1μm is used for all membranes because the selective layer thicknesses are unknown. Examples of how this equation can be used to describe the permeability of different types of membranes are provided below.

### Process calculations

The dehydration of ethanol by PV in a single-pass continuous flow process will be the model system in this work, as it was in the earlier review article.^2^ The reduction in water content from 10 to 1 wt % for a continuous feed flow rate of $0.0556\;\text{kg}{\;s}^{-1}\left(0.05\;\text{kg}{\;s}^{-1}\right.$ ethanol) will serve as the reference process. These are the same flow rate and feed concentration that were used in the previous review article^2^ and they represent the daily processing of 5400 L of ethanol (2 million liters per year). The baseline scenario in the previous review article involved the reduction of water to 0.5 wt %, but this would extend below the range of water concentrations for reliable performance data for the membranes involved in this work. Thus, a final water content of 1 wt% was selected here. The 10 to 1 wt% range in water concentration highlights the ability of PV to cross the ethanol/water azeotrope (4.0 wt% water at 101 kPa and 78.0 °C) and it overlaps with the ranges of water concentrations available from the literature performance data sets used herein. In addition, a water concentration of 1 wt% is relevant because it is the level to which many solvents must be dried to retain their solvent power.^48^ The relative volatility of water and ethanol in this concentration range is close to 1, making standard distillation highly inefficient, and it is equal to 1 at the azeotrope, making standard distillation impractical.^2^ Thus, alternatives to standard distillation are required not only to cross the azeotrope but to approach the azeotrope and dry the ethanol beyond it. The earlier review article included calculations for the same range of water concentrations and for an ethanol feed flow rate of 0.05 kg s^−1^, although the benchmark for calculations in that work was water reduction from 10 to 0.5 wt%^2^

As detailed in the previous review article, the spreadsheet calculators divide a dehydration membrane system into 100 sub-units of either 100 area increments (for PV or V·P single-pass continuous process calculations) or 100 time increments (for PV batch process calculations). Increasing the number of sub-units to 1000 in the earlier work did not significantly change the calculated values and so 100 sub-units are also used in this work. In each sub-unit, the number of moles of water and solvent transferred across the membrane in that sub-unit are calculated as follows:

(11) $$\text{Single-Pass}\;\text{PV}\;\text{Process}\;\text{Sub-units}:{\left[\Delta {\dot{N}}_{i}\right]}_{k}={\left[-j_{i}\right]}_{k}A_{k}={\left[-\frac{P_{i}}{\ell }\left(x_{i}\gamma_{i}p_{i}^{sat}-y_{i}p^{P}\right)\right]}_{k}A_{k}$$

(12) $$\text{Single-Pass}\;\text{V}\cdot \text{P}\;\text{Process}\;\text{Sub-units}\;:{\left[\Delta {\dot{N}}_{i}\right]}_{k}={\left[-j_{i}\right]}_{k}A_{k}={\left[-\frac{P_{i}}{\ell }\left(x_{i}p^{F}-y_{i}p^{P}\right)\right]}_{k}A_{k}$$

(13) $$\begin{array}{l} \text{Batch}\;\text{PV}\;\text{Process}\;\text{Sub-units}\;:\;{\left[\Delta N_{i}\right]}_{k}\\ =A{\left[-j_{i}\right]}_{k}\Delta t_{k}\\ =A{\left[-\frac{P_{i}}{\ell }\left(x_{i}\gamma_{i}p_{i}^{sat}-y_{i}p^{P}\right)\right]}_{k}\Delta t_{k} \end{array}$$

where k is the sub-unit number and $\Delta N_{i}$ and $\Delta {\dot{N}}_{i}$ are the number of moles of compound i and the molar rate of compound i transferred across the membrane in the sub-unit, respectively. For single-pass systems, $A_{k}$ is the area of the sub-unit. For batch systems, the membrane area in the system is A and $\Delta t_{k}$ is the time interval for the sub-unit. Permeate mole fractions $\left(y_{i}\right)$ are calculated for each sub-unit. The total permeate stream flow and properties are determined by summing the permeate flows from the individual sub-units. Because the amount of each component permeating the membrane in a sub-unit is subtracted from the feed for that sub-unit to arrive at the feed to the next sub-unit, component balances between the feed (or initial batch), retentate (or final batch), and permeate are inherently closed. In the previous article, permeability in the sub-unit expressions was assumed to be constant. In this work, it is calculated based on the temperature of the feed fluid entering a sub-unit and the average feed-side activity within a sub-unit, according to an expression based on Eqn (4). In a single-pass system, the retentate from one area sub-unit becomes the feed for the next area sub-unit. In a batch system, the permeate from one time sub-unit is subtracted from the bulk mixture to calculate the feed mixture to the next time sub-unit. Aside from the change in how permeability is determined, the process calculators in this work are the same as those used in the previous work. The calculators are prepopulated with the necessary thermodynamic and physical property parameters for the following solvents and their binary mixtures with water: acetone, acetonitrile, 1-butanol, *N,N*-dimethyl acetamide (DMAC), *N,N*-dimethyl formamide (DMF), ethanol, methanol, methyl isobutyl ketone (MIBK), methyl tert-butyl ether (MtBE), *N*-methyl-2-pyrrolidone (NMP), 2-propanol (IPA), and tetrahydrofuran (THF). Other solvents can be incorporated into the spreadsheet calculators by providing the necessary parameters in the ‘User Solvent’ row of the ‘Properties’ sheet.

The impact of using a variable permeability in these calculations will be explored using the properties of three membranes: a NaA zeolite membrane and two PVA membranes with different degrees of crosslinking.

### Example permeance expressions: NaA membrane

**Figure 3.**
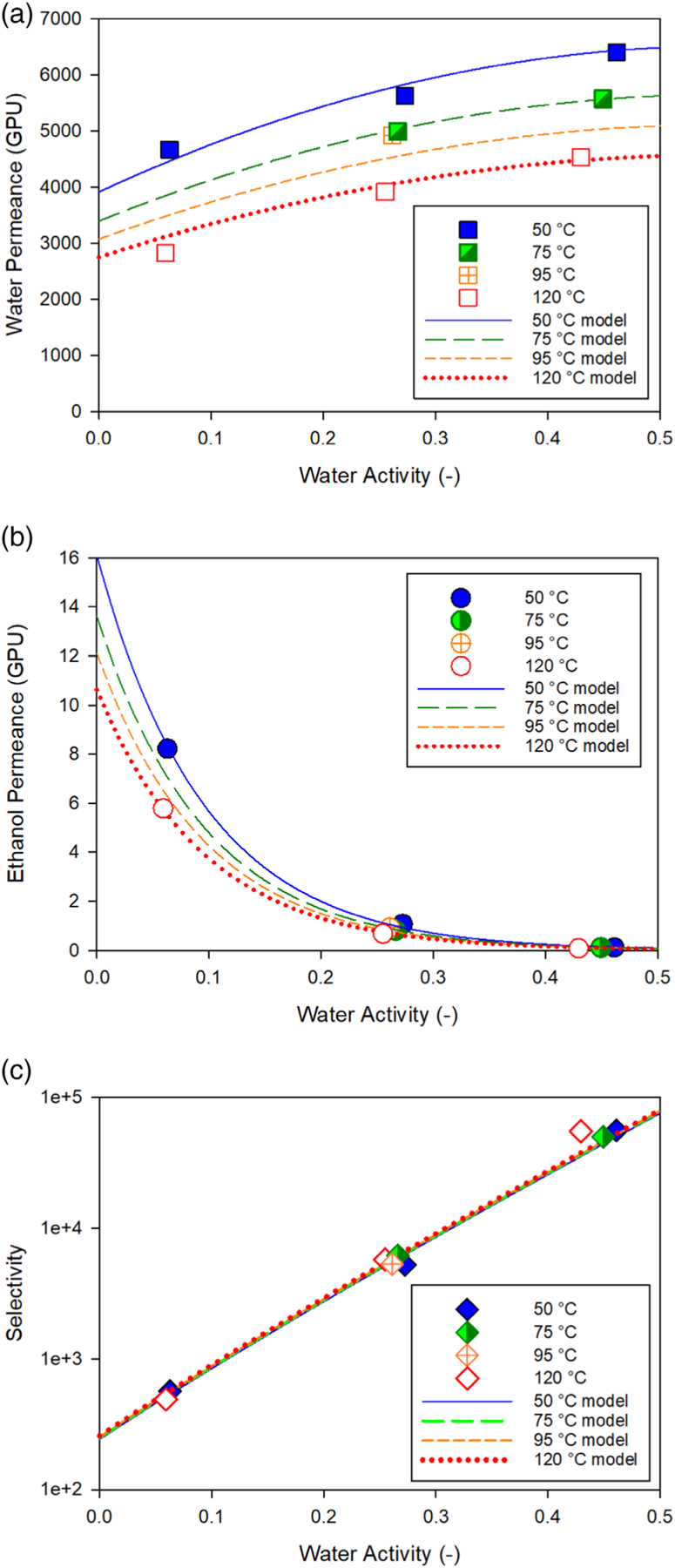
(a–c): Water permeance (a), ethanol permeance (b), and water/ethanol selectivity (c) as a function of feed-side water activity for NaA membrane. Symbols represent values calculated from PV performance data presented by Kondo *et al*.^11^ Lines represent the result of fitting the experimental results to permeability expression Eqn (4) (coefficients listed in Table 1). The experimental data and calculated values from Kondo *et al*. are provided in Table S5 of Supporting Information.

Kondo et al. reported bench-scale PV performance data for a NaA zeolite membrane separating ethanol/water mixtures by PV as a function of water concentration and temperature.^11^ The original data, calculated activities, and calculated permeances are presented in Table S5 of the Supporting Information. The variation of water and ethanol activities with water concentration in the feed-side liquid (as weight %) is illustrated for a temperature of 95 °C in Fig. S1 of the Supporting Information. The water permeances, ethanol permeances, and water/ethanol selectivities calculated for data in the water concentration range of 1 to 10 wt% (corresponding to water activities of 0.06 to 0.44) and the temperature range of 50 to 120 °C are presented as symbols in Fig. 3(a)–(c), respectively. A total of nine data points were available in this range. As detailed in Section 2 of the Supporting Information, the following forms of Eqn (4) were developed to describe the water and ethanol permeances for this data set:

(14) $$\Pi_{w}\left(\text{GPU}\right)=3914.0\;\text{exp}\left[\left(\frac{-5330.1}{8.314}\right)\left(\frac{1}{323.15\text{ K}}-\frac{1}{T}\right)\right]\;\left(1+2.3753a_{w}-2.1212a_{w}{\;}^{2}\right)$$

(15) $$\Pi_{e}\left(\text{GPU}\right)=16.082\;\text{exp}\left[\left(\frac{-6250.7}{8.314}\right)\left(\frac{1}{323.15\text{ K}}-\frac{1}{T}\right)\right]\exp\left(-10.46a_{w}\right)$$

A default membrane thickness of 1μm was assumed to convert the 3914.0 and 16.082 GPU pre-exponential values in Eqns (14) and (15) to $P_{ow}=1.3090\times 10^{-12}\text{kmol}\;m\;m^{-2}{\;s}^{-1}{kPa}^{-1}$ and $P_{oe}$ $=5.3787\times 10^{-15}\text{kmol}\;m\;m^{-2}{\;s}^{-1}{kPa}^{-1}$, respectively, for use in the permeability expression of Eqn (4). The full set of values used in Eqn (4) are provided in Table 1. As indicated, a reference temperature of 50 °C(323.15 K) was used in the Arrhenius term. These relationships are plotted in Fig. 3(a), (b) as isotherms at the experimental temperatures of 50, 75, 95, and 120 °C. The resulting selectivity isotherms from these permeance relationships are shown in Fig. 3(c).

For this membrane, water permeance is represented by a quadratic expression in terms of water activity, while ethanol permeance is represented by an exponential expression in terms of water activity. As indicated in Fig. 3(a), (b), water permeance increases modestly with increasing water activity, but ethanol permeance decreases rapidly with increasing water activity. This combination results in a selectivity that rises rapidly with increasing water activity — by 2 orders of magnitude as water content increases from 1 to 10 wt% (an increase in water activity from 0.06 to 0.44). Such a dramatic change in ethanol permeance at low water concentrations has been attributed to the interaction of water with non-zeolitic pathways in the selective layer.^14,15^ Both permeances decrease with increasing temperatures, as indicated by negative Arrhenius parameters $\left(E_{i}\right)$. The decline in both permeances with increasing temperature is relatively small and of a similar magnitude, resulting in a selectivity that does not change much with temperature, as indicated by the clustering of selectivity values from the experimental data and the overlapping model lines in Fig. 3(c).

### Example permeance expressions: poly(vinyl alcohol) membranes

A recent paper from the PV/V⋅P system and membrane manufacturer Deltamem AG (Switzerland) provides performance data for two types of commercial PVA-based composite membranes PERVAP^™^ 4100 and PERVAP^™^ 4101 (herein, ‘PVA 4100’ and ‘PVA 4101’).^6^ The membranes consist of a hydrophilic PVA selective layer coated on a porous support. The two membranes differ primarily in the degree of crosslinking in the PVA layer, with the PVA layer of the PVA 4101 being more crosslinked than that of the PVA 4100. Greater crosslinking reduces swelling when in contact with water which, in turn, makes the properties of the membrane more stable when the concentration of water changes. The recommended maximum long-term operating temperature for these membranes is 100 °C.^49^

**Figure 4.**
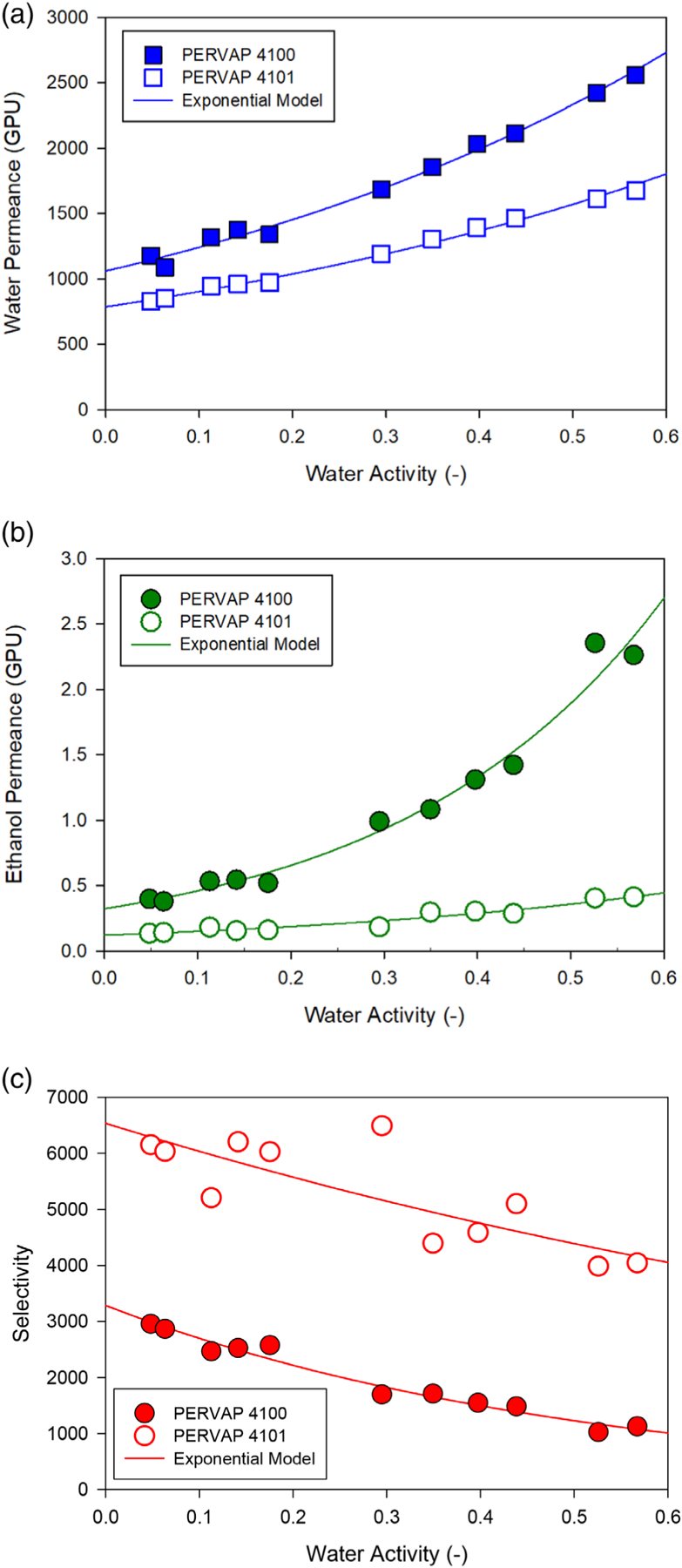
(a–c): Water permeance (a), ethanol permeance (b), and water/4100 and 4101 membranes. Symbols represent values calculated from PV performance data presented by Yave.^6^ Lines represent the result of fitting the experimental results to permeability expression Eqn (4) (coefficients listed in Table 1). The experimental data and calculated values from Yave are provided in Tables S7 and S8 of Supporting Information.

The original data and calculated permeances are presented in Tables S7 and S8 of the Supporting Information. The water permeances, ethanol permeances, and water/ethanol selectivities calculated for data in the water concentration range of 0.79 to 15.4 wt% (corresponding to water activities of 0.048 to 0.57) at a temperature of 95 °C are presented as symbols in Fig. 4(a)–(c), respectively. A total of 11 data points were available in this concentration range. The temperature exponential constant for the water permeance $\left(E_{w}\right)$ was determined from the flux data provided at 60 and 105 °C with 10 wt% water in the ethanol feed for each PVA membrane, as reported in the aforementioned journal article from Deltamem AG^6^ and as detailed in Table S6 and Section 3 of the Supporting Information. Presumably, the testing at 105 °C was of a sufficiently short duration to avoid any potential long-term effects of operating over 100 °C. The same type of information was not provided for ethanol flux, so the temperature exponential for ethanol permeance $\left(E_{e}\right)$ could not be directly calculated. For the purposes of this work, $E_{e}$ will be assumed to be the same as $E_{w}$. Due to this assumption, analysis of the effect of temperature changes on the process will be limited to the membrane area under ideal conditions (i.e., zero permeate pressure and isothermal operation) — conditions under which membrane area is almost wholly dependent on water permeance. The following correlations were developed to describe the water and ethanol permeances for the PVA 4100 and 4101 membranes:

(16) $$\begin{array}{l} \text{ PVA }4100:\Pi_{w}\left(\text{GPU}\right)\\ =1061.0\;\text{exp}\left(1.5771a_{w}\right)\text{exp}\left[\left(\frac{15210}{8.314}\right)\left(\frac{1}{368.15\text{ K}}-\frac{1}{T}\right)\right] \end{array}$$

(17) $$\begin{array}{l} \text{PVA }4100:\Pi_{e}\left(\text{GPU}\right)\\ =0.32289\;\text{exp}\left(3.5393a_{w}\right)\text{exp}\left[\left(\frac{15210}{8.314}\right)\left(\frac{1}{368.15\text{ K}}-\frac{1}{T}\right)\right] \end{array}$$

(18) $$\begin{array}{l} \text{PVA }4101:\Pi_{w}\left(\text{GPU}\right)\\ =788.02\;\text{exp}\left(1.3796a_{w}\right)\text{exp}\left[\left(\frac{22530}{8.314}\right)\left(\frac{1}{368.15\text{ K}}-\frac{1}{T}\right)\right] \end{array}$$

(19) $$\begin{array}{l} \text{ PVA }4101:\Pi_{e}\left(\text{GPU}\right)\\ =0.12056\;\text{exp}\left(2.1748a_{w}\right)\text{exp}\left[\left(\frac{22530}{8.314}\right)\left(\frac{1}{368.15\text{ K}}-\frac{1}{T}\right)\right] \end{array}$$

The thickness of the selective layer was not provided, so a default membrane thickness of 1μm was assumed to convert the preexponential values to permeabilities for use in the permeability expression of Eqn (4). The values used in Eqn (4) for the PVA membranes are provided in Table 1. As indicated, a reference temperature of 95 °C (368.15 K) was used in the Arrhenius term because this was the temperature of the main concentration dependency experiments. Eqns (16)–(19) are plotted in Fig. 4(a), (b) along with the 95 °C data as a function of water activity. The resulting selectivities from these permeance relationships are shown in Fig. 4(c).

All the permeance expressions for the PVA membranes have an exponential relationship with respect to water activity and these coefficients are greater than zero, resulting in permeances that increase with water activity. The exponential coefficient for ethanol permeance is larger than that for water, indicating that ethanol permeance increases more rapidly than water permeance as water activity is increased. By contrast, the exponential coefficient in the ethanol permeance expression for the NaA membrane was less than zero and several times the magnitude of those for the PVA membranes. The positive value of $E_{w}$ for the PVA membranes indicates that water permeance increases with increasing temperature — opposite the behavior of the NaA membrane.

For this work, the water and ethanol permeances of each membrane are assumed to be represented by the permeability expressions shown above (Eqns (14)–(19)) with no errors associated with those parameters. The experimental data used to determine permeability parameters are subject to experimental errors that will, in turn, lead to uncertainty in the parameters. The curve fitting process to calculate the parameters will generate standard errors for each parameter that may, or may not, capture the extent of the potential experimental error. For example, the relative standard error for the water permeability pre-exponential parameter, $P_{ow}$, from the curve fitting process was 8.7, 2.1, and 1.2% for the NaA, PVA 4100, and PVA 4101 membranes, respectively, while the relative standard error for $P_{oe}$ ranged from 4 to 7%. The impact of uncertainty in the permeability parameters can be explored using the process calculators by perturbing the membrane performance parameters (based on either known experimental errors or curve fitting errors) and observing the change in predicted process performance (i.e., membrane area, permeate composition). This type of analysis is outside the scope of this work but may be useful to researchers or system designers considering the uncertainty in process performance that can result from uncertainty in membrane performance.

## MEMBRANE SYSTEM DESIGN

The differences in the response of permeance to water concentration and temperature between the three membranes will be used to explore the impact of including variable permeability into PV and V⋅P design calculations. As shown in Fig. 2, the ‘baseline’ scenario is the drying with a single-pass PV system of an ethanol/water mixture from 10 wt% to 1 wt% ($x_{w}$ from 0.2213 to 0.02518) for a feed liquid consisting of 0.05 kg s^−1^ of ethanol under isothermal conditions at 95 °C (with 10 wt% of water in the feed, the total feed flow rate is 0.0556 kg s^−1^). Under these feed conditions, water and ethanol activities are 0.438 are 0.802, respectively. In the 1 wt% water retentate leaving the process, these activities become 0.0604 and 0.978.

### Effect of composition on permeances and driving force

The permeances and selectivities for the three membranes under the baseline feed and retentate conditions, as calculated from the permeance expressions, are listed in Table 2. For all three membranes, performance under the feed composition is significantly different than that at the retentate composition. For example, water permeance at 1 wt% water is lower than that at 10 wt% for all three membranes, most noticeably for the less-crosslinked PVA 4100, which exhibited a 45% reduction in $\Pi_{w}$ from the feed to retentate conditions. The relative change in ethanol permeance was more pronounced than the change in water permeance for the membranes and the NaA and PVA membranes behaved quite differently when it came to ethanol permeance. Ethanol permeance of the NaA membrane was dramatically (52 times) higher at 1 wt% water than at 10 wt% water, while that of the PVA membranes was reduced at the lower water concentration by 56 to 74% relative to the ethanol permeance at 10 wt% water.

**Figure 5.**
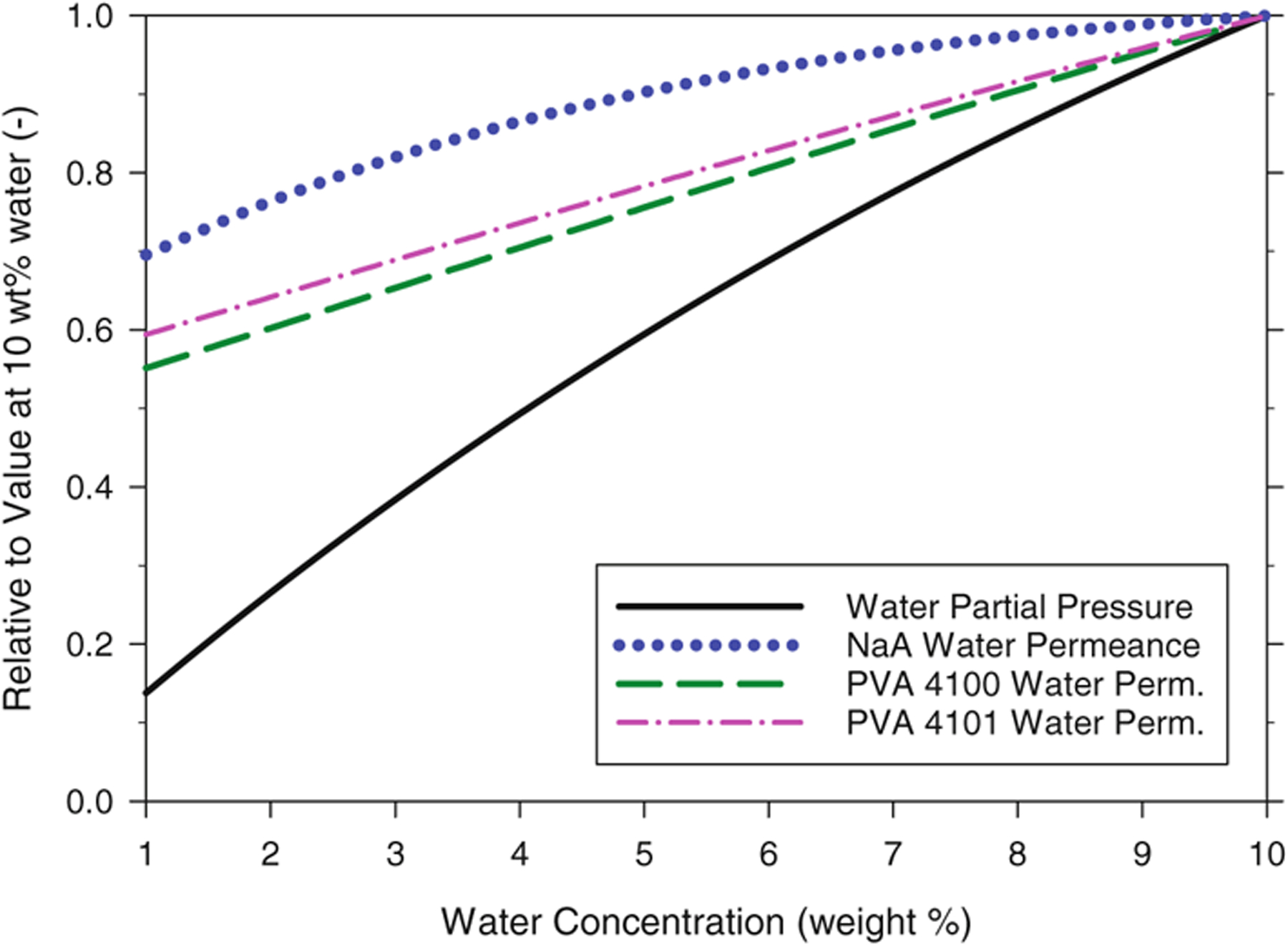
Effect of feed-side water concentration on the relative water permeances of the membranes and on the relative partial vapor pressure of water. Each value has been normalized by the value at a water concentration of 10 wt% (as listed in Table 2) and was calculated with single-pass PV spreadsheet using variable permeabilities under the following baseline conditions: isothermal operation at 95 °C, 0.05 kg s^−1^ ethanol feed rate, 0 kPa permeate pressure. Water partial pressure at 10 wt% water and 95 °C is 37.0 kPa.

**Figure 6.**
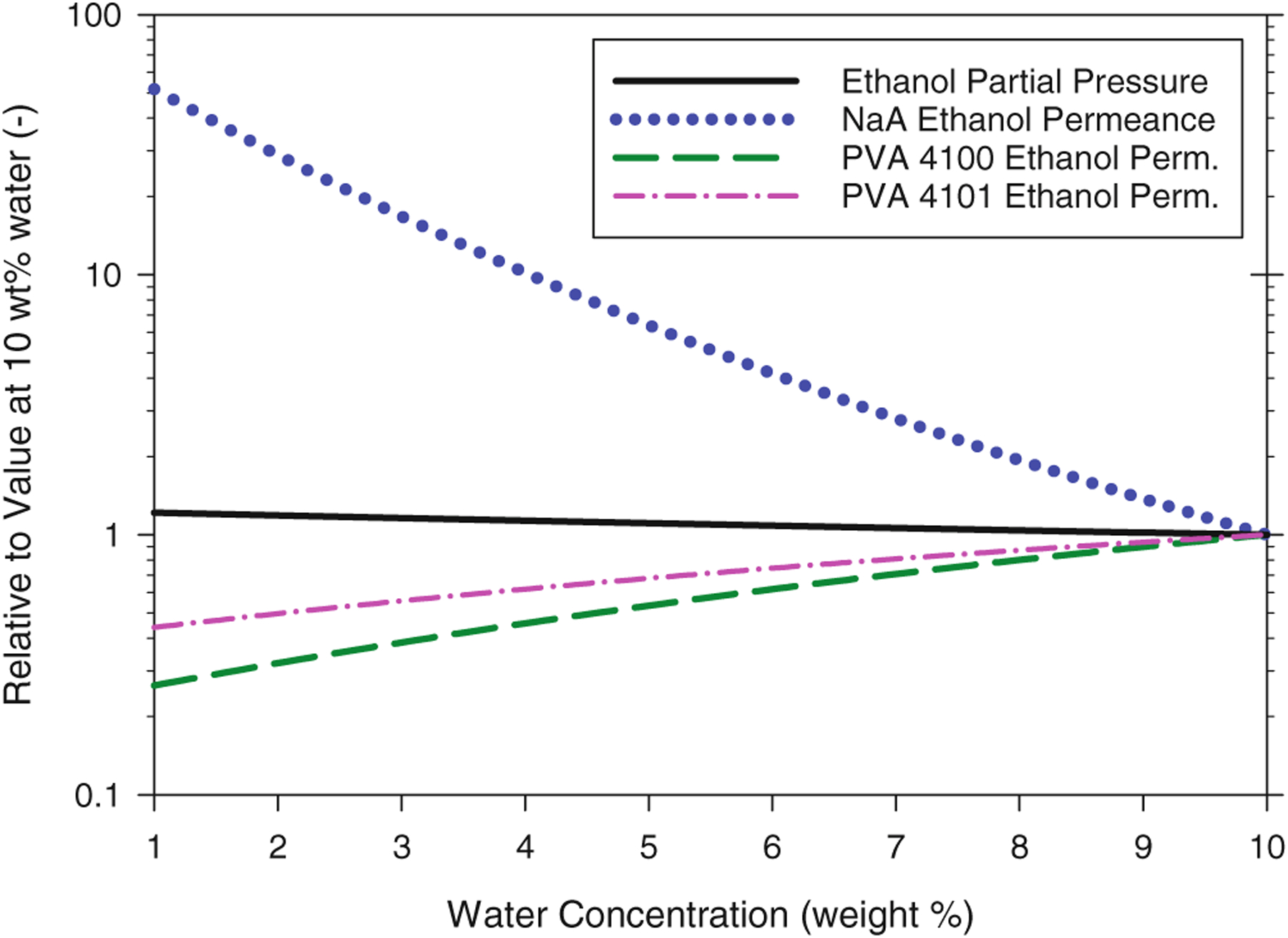
Effect of feed-side water concentration on the relative ethanol permeances of the membranes and on the relative partial vapor pressure of ethanol. Each value has been normalized by the value at a water concentration of 10 wt% (as listed in Table 2) and was calculated with single-pass PV spreadsheet using variable permeabilities under the following baseline conditions: isothermal operation at 95 °C, 0.05 kg s^−1^ ethanol feed rate, 0 kPa permeate pressure. Ethanol partial pressure at 10 wt% water and 95 °C is 151.7 kPa.

The effects of water concentration on the water permeances of the three membranes and on the water partial vapor pressure for an ethanol/water mixture at 95 °C, normalized by the value at 10 wt% water, are shown in Fig. 5. Since all values have been normalized by the respective value at 10 wt% water, each curve in the figure has a value of 1.0 at 10 wt% water. An equivalent graph for relative ethanol permeances and relative ethanol partial vapor pressure is provided in Fig. 6. As indicated in Fig. 5, the partial pressure of water (solid line) changes by almost an order of magnitude as water in the feed-side liquid falls from 10 wt% to 1 wt%. The relative water permeances also decrease for each membrane as the water concentration in the feed decreases, but not as much as water partial pressure decreases. As governed by Eqn (10), when permeate pressure is negligible, species flux is equal to the product of permeance and feed-side partial pressure. Thus, water flux for the membranes will decline as feed-side water concentration decreases, driven mostly by the falling partial pressure of water, but also by the falling water permeance.

By contrast, as indicated by the solid line in Fig. 6, the partial pressure of ethanol *increases* modestly (+22%) as the water concentration in the feed-side fluid decreases from 10 wt% to 1 wt% due to the rising mole fraction of ethanol in the feed fluid (note the log scale for the y-axis in Fig. 6). Whereas changes in water flux were associated with changes in both permeance and partial pressure, changes in ethanol flux due to feed composition changes will be controlled almost entirely by changes in permeance, as indicated by the sizeable changes in relative ethanol permeances compared to those of relative ethanol partial pressure. As noted earlier, the response of ethanol permeance of the NaA membrane to changes in feed-side water concentration is dramatic and opposite to that of the PVA membranes. This is clearly shown in Fig. 6 by the divergence between the NaA and PVA relative permeance lines as water concentration decreases from 10 wt%.

### Effect of retentate water concentration on membrane area and ethanol recovery

**Figure 7.**
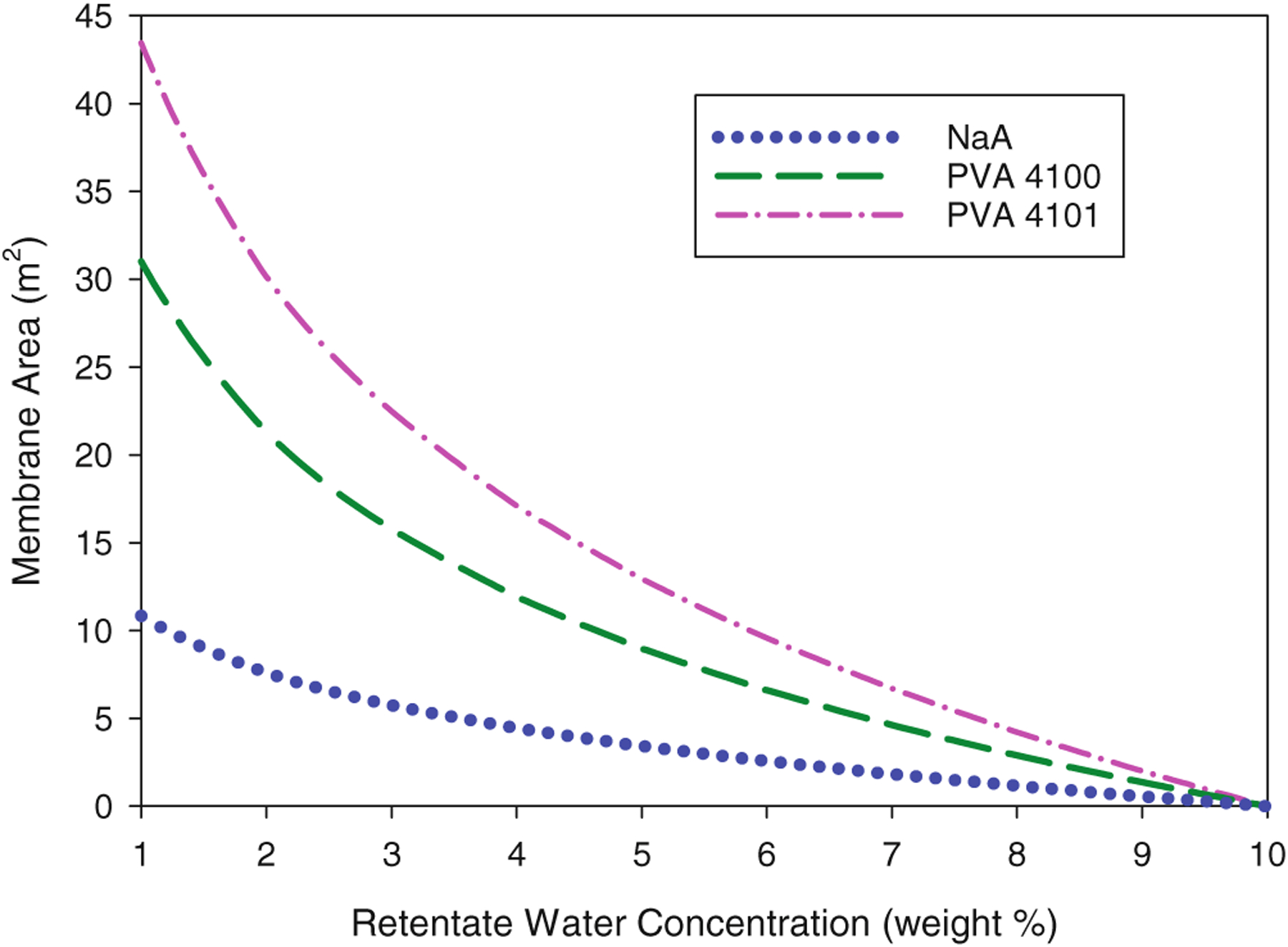
Membrane area of a single-pass PV system based on the NaA or PVA membranes removing water from a feed stream containing 10 wt% water as a function of the water content in the retentate stream. Values were calculated with single-pass PV spreadsheet using variable permeabilities under the following baseline conditions: isothermal operation at 95 °C, 0.05 kg s^−1^ ethanol feed rate, 0 kPa permeate pressure.

**Figure 8.**
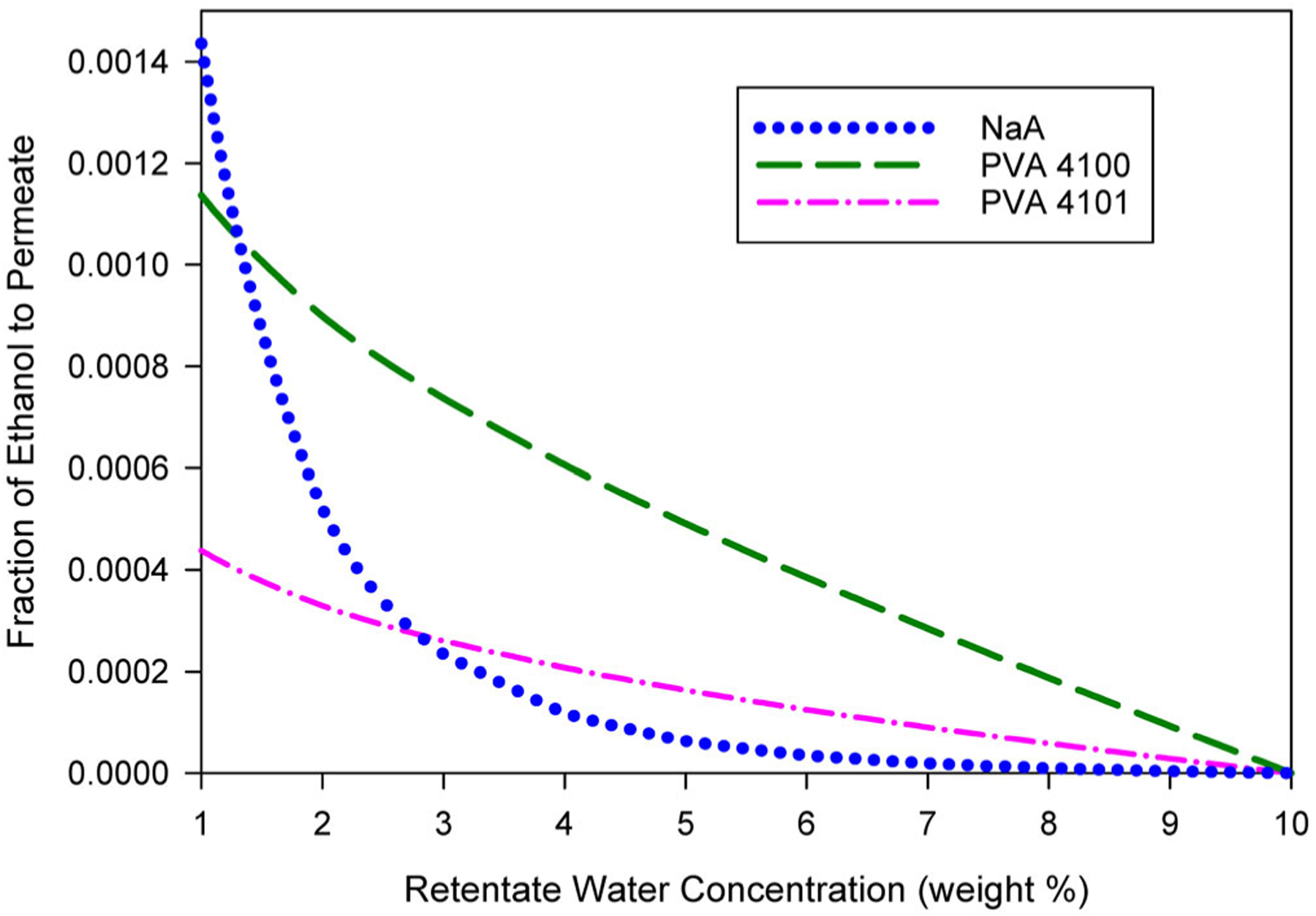
The fraction of ethanol from the feed stream that is transferred to the permeate stream for the conditions of Fig. 7.

Using the permeance relationships established above, the single-pass PV spreadsheet calculator was used to predict the membrane area required to reduce the water concentration from the baseline feed of 10 wt% as a function of the final retentate water concentration. The areas calculated for the three membranes are shown in Fig. 7. The baseline scenario of a 1 wt% water retentate is represented by the y-intercept of each curve. Single-pass PV systems based on the NaA, PVA 4100, and PVA 4101 membranes are estimated to require 10.8, 31.0, and 43.4m^2^, respectively, to reduce water from 10 wt% to 1 wt% for the baseline assumptions. For each membrane, about half of the area is needed to reduce water from 10 wt% to 3 wt%. The other half of the membrane area reduces water from 3 wt% to 1 wt%. The reason the second half of the area only removes 20–25% of the water is due to both decreasing water driving force and decreasing water permeance as the feed-side water concentration decreases. Further reductions in the retentate water concentration would require ever larger relative amounts of additional membrane area. In this work, the minimum retentate water content that can be modeled is limited by the range in water concentration of the membrane performance data sets. However, the effect of varying the retentate water concentration to as low as 0.1 wt% on membrane area was explored for a hypothetical membrane with constant permeances in the previous review article (presented in Fig. 8 of the reference).^2^

The fraction of ethanol transferred from the feed to the permeate for the same conditions as those for Fig. 7 are shown in Fig. 8. As the retentate water concentration decreases, the amount of ethanol lost to the permeate increases. An increase in ethanol transfer to the permeate is expected because the membrane area of the system increases as retentate water concentration decreases. As indicated by the y-intercepts of the curves, the fraction of ethanol in the feed that is transferred to the permeate for single-pass PV systems reducing water from 10 wt% in the feed to 1 wt% in the retentate based on the NaA, PVA 4100, and PVA 4101 membranes is predicted to be $1.44\times 10^{-3},1.14\times 10^{-3}$, and $0.44\times 10^{-3}$, respectively. These correspond to ethanol recoveries in the retentate of 99.86%, 99.89%, and 99.96% and average concentrations of ethanol in the permeate of 1.4, 1.1, and 0.43 wt%, respectively. Thus, the amount of ethanol lost to the permeate is expected to be small for all three membranes, although there are distinct differences between the behavior of the NaA membrane and the PVA membranes.

Ethanol transferred to the permeate through the PVA membranes increases relatively linearly with decreasing retentate concentration over the entire 10 to 1 wt% range. By contrast, the amount of ethanol permeating the NaA membrane grows exponentially as retentate water concentration is decreased. For a system based on the NaA membrane with 1 wt% water in the retentate, 85% of ethanol transfer to the permeate occurs in the section of the system reducing water from 3 wt% to 1 wt%. For that section, the permeate concentration of ethanol would be 5.5 wt%, which is much higher than the 0.29 wt% ethanol in the upstream section reducing water from 10 wt% to 3 wt%. In such a case, it might be advantageous to collect separate permeate streams from the two sections, rather than one combined stream. Such a decision would depend on ethanol recovery targets and permeate processing options/requirements.

### Variable vs. fixed permeances

**Figure 9.**
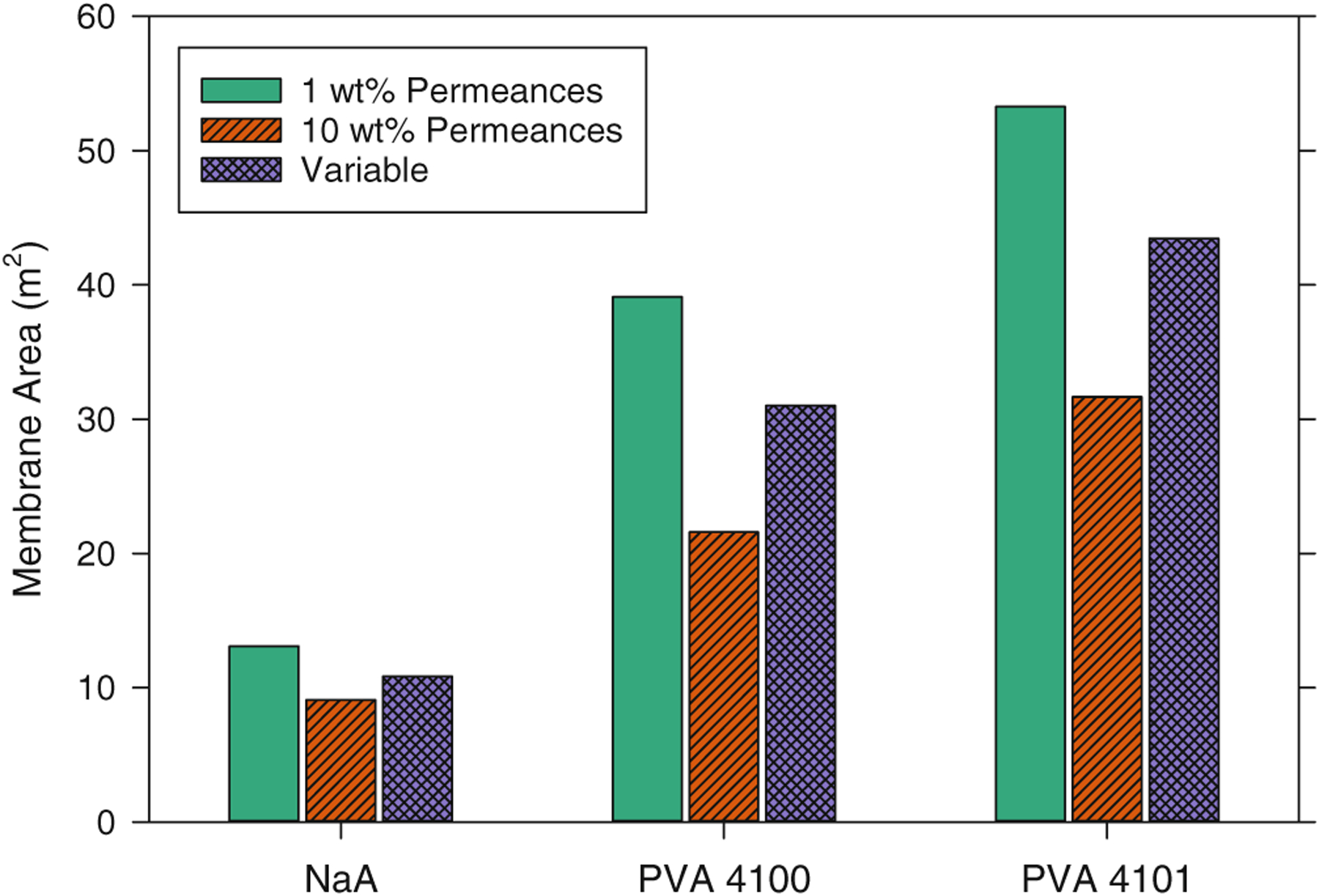
Membrane area required to change water concentration in ethanol from 10 wt% to 1 wt% for a continuous PV system with the NaA or PVA membranes assuming either variable or fixed permeances. The bars (L to R) for each membrane represent permeances fixed at those corresponding to 1 wt% water, fixed at 10 wt% water, and variable according to the expressions established for each. Values were calculated with single-pass PV spreadsheet under the following baseline conditions: isothermal operation at 95 °C, 0.05 kg s^−1^) ethanol feed rate, 0 kPa permeate pressure.

To investigate the differences between system performance estimates made with fixed permeances and estimates based on variable permeances, single-pass PV calculations were made for the baseline change in water concentration from 10 to 1 wt%, but with permeances fixed at either the values corresponding to the 1 wt% water retentate or the values corresponding to 10 wt% water feed (as listed in Table 1). The results are shown in Fig. 9 for the NaA, PVA 4100, and PVA 4101 membranes, along with the membrane areas calculated with the variable permeance expressions for comparison. Water permeances fixed at 1 wt% and 10 wt% water values represent the minimum and maximum, respectively, for each membrane over the baseline composition range. As a result, the membrane area required to achieve the desired water removal is greatest when permeances are assumed to be fixed at 1 wt% values and smallest when fixed at 10 wt% values. The areas for the fixed permeance cases for all the membranes differed by 20–30% from those calculated with variable permeances. Thus, for the membranes and conditions covered in this work, the inclusion of permeances that vary with the feed-side composition is expected to have a noticeable effect on the predicted membrane area requirement.

**Figure 10.**
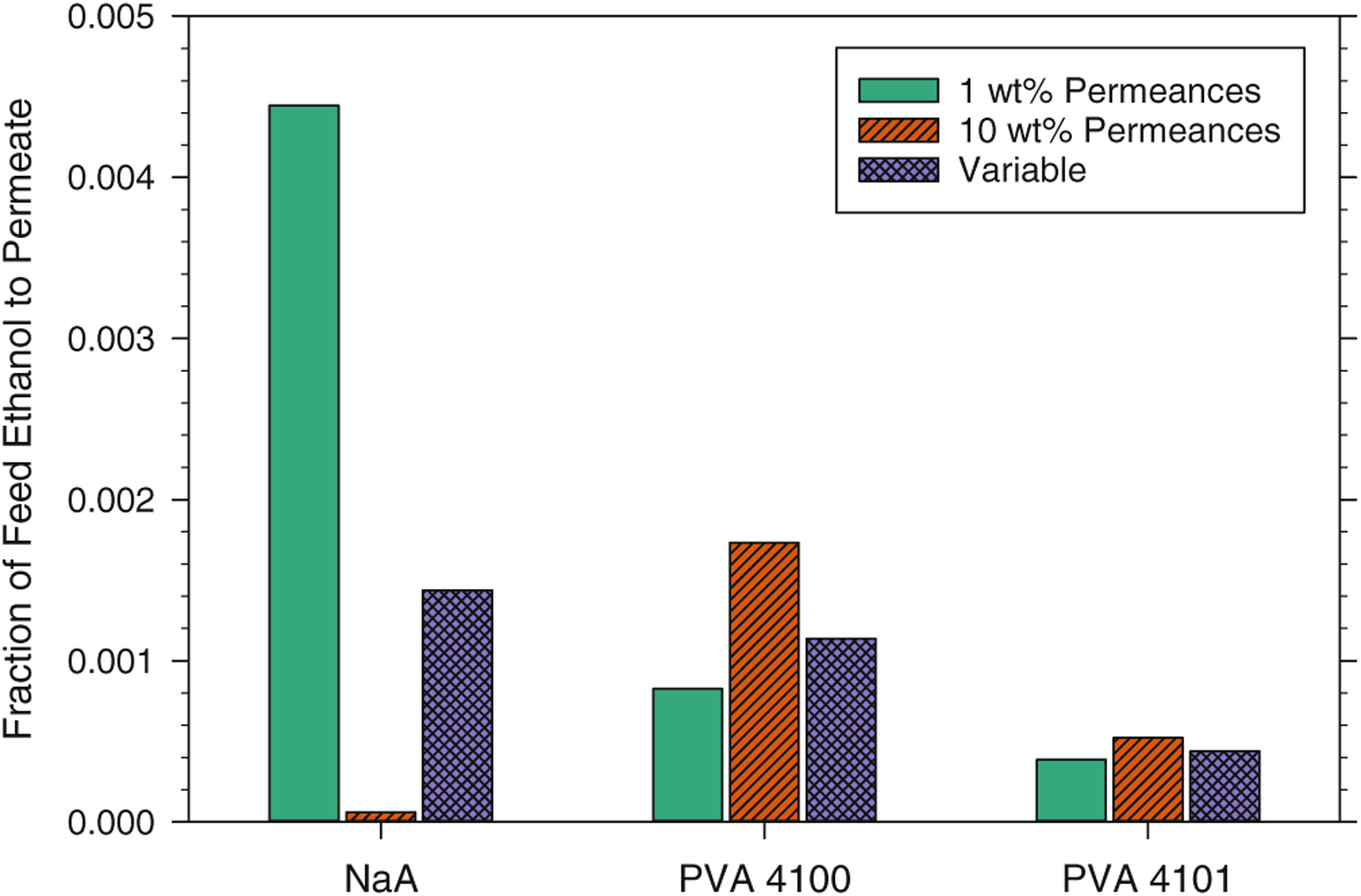
The fraction of ethanol from the feed stream that is transferred to the permeate stream for the conditions stated for Fig. 9.

Another measure of the impact of fixed vs. variable permeances is the amount of ethanol transferred from the feed to the permeate. For the fixed permeance and variable permeance cases described above, the fraction of ethanol in the feed of the system transferred to the permeate for each membrane is shown in Fig. 10. As described earlier, and as shown in Fig. 6, the ethanol permeance of the NaA membrane is highly dependent on the feed-side water concentration with high ethanol permeance at low water concentrations. Thus, the assumption of permeances fixed at the 1 wt% water value for the NaA membrane will result in predictions of large transfers of ethanol to the permeate that are about three times the value predicted with variable permeances. This is represented by the largest bar in Fig. 10. Conversely, small transfers are predicted for the NaA membrane with permeances fixed at the 10 wt% values — a factor of 24 times lower than the variable permeance case — as represented by the smallest bar in Fig. 10. Because ethanol permeances for the PVA membranes behave in the opposite manner to that of the NaA membrane by decreasing with decreasing water concentration, ethanol transfer to the permeate for the PVA membranes will be lower if permeances are fixed at the 1 wt% water values compared to variable permeances and they will be higher when permeances are fixed at the 10 wt% water values — opposite the trend of the NaA membrane. In addition, because the ethanol permeances of the PVA membranes do not change as much with water concentration as that of the NaA membrane, the differences between ethanol losses for the fixed permeance and variable permeance cases for the PVA membranes are smaller for the PVA membranes than for the NaA membrane. Thus, as with membrane area requirement, the inclusion of permeances that vary with the feed-side composition is expected to have a noticeable effect on the predicted ethanol losses to the permeate stream, at least for the membranes and conditions covered in this work.

**Figure 11.**
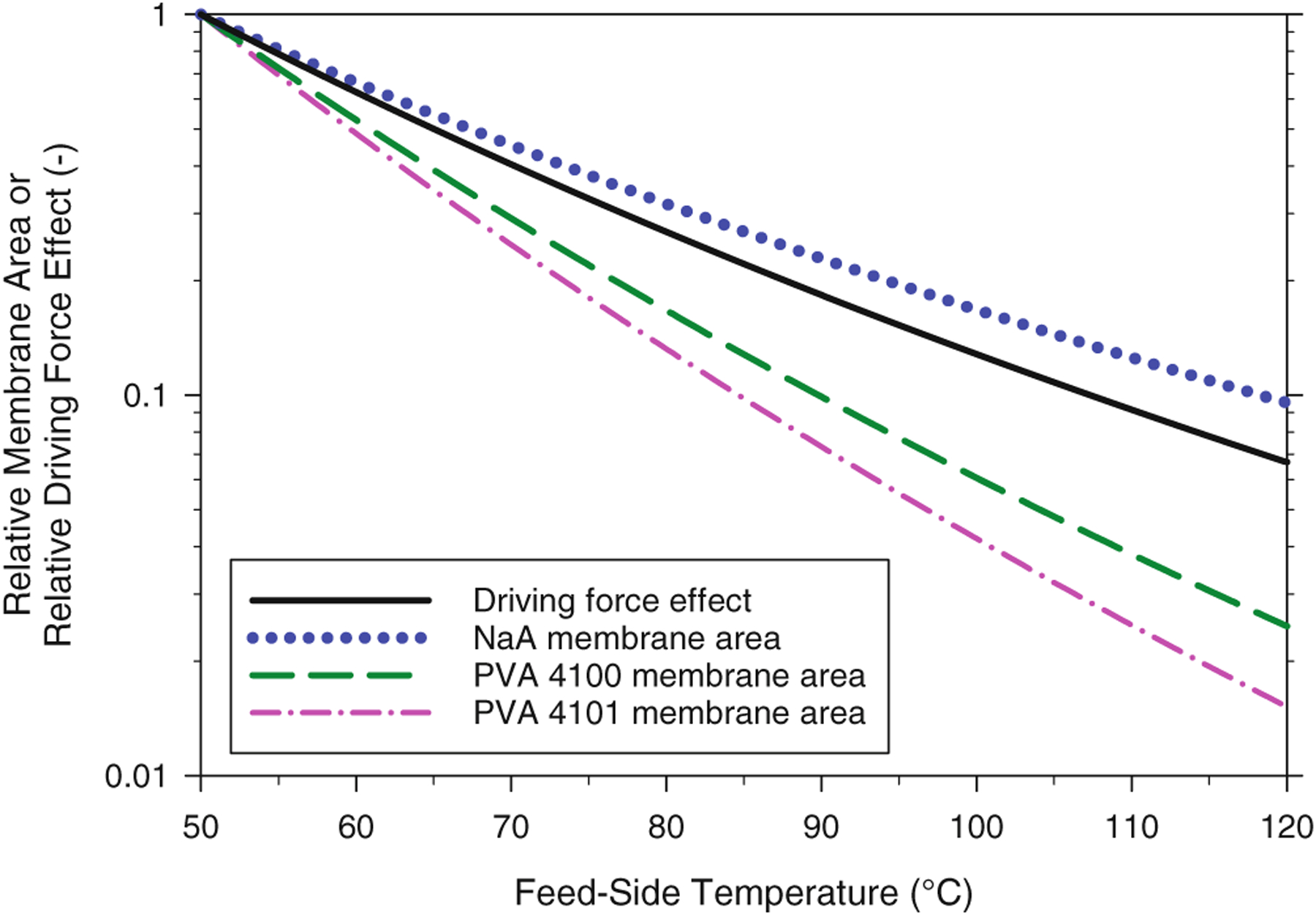
Effect of feed temperature on the relative membrane area required to reduce water in ethanol from 10 wt% to 1 wt% compared to the relative membrane area if only the driving force is considered. The relative area is the ratio of the membrane area predicted at the given temperature to the membrane area calculated with a feed temperature of 50 °C. The driving force effect is the ratio of the water vapor pressure at 50 °C to the vapor pressure of water at a given temperature.

As noted above, the feed-side temperature in PV systems significantly affects the partial pressure driving force for both ethanol and water. Since vapor pressures increase nearly exponentially with increasing temperature, higher temperatures will yield large increases in the vapor pressure driving force for transport, which, in turn, will dramatically decrease the amount of membrane area required for a desired extent of water removal. The relative effect of increased vapor pressure on membrane area is depicted in Fig. 11 as a solid line. This line represents the membrane area required for the baseline separation (*i.e.*, 10 wt% water feed to 1 wt% water retentate) for isothermal operation at a given temperature, relative to the area required for a system operating at 50 °C. This line represents the relationship between membrane area and temperature for membranes with temperatureindependent permeances. Thus, for such membranes in the baseline separation scenario, the membrane area required for a 120 °C system would be 93.3% lower than for the same membrane operating at 50 °C.

Based on the permeability expressions for the NaA and PVA membranes, the water permeances are temperature-dependent. For the NaA membrane, increasing temperature results in a decreased water permeance ($E_{w}$ in Eqn (4) is negative), whereas the opposite was observed for the PVA membranes ($E_{w}$ is positive). Thus, the NaA membrane became less efficient at removing water at higher temperatures, while PVA membranes became more efficient. Membrane areas for the baseline separation were calculated for each membrane at temperatures ranging from 50 to 120 °C. The areas, normalized by the area for a 50 °C system, are shown in Fig. 11. As shown, the reduction in area due to increasing temperature is better than the vapor pressure driving force effect for the PVA membranes, but worse than the driving force effect for the NaA membranes, reflecting the relative changes in water permeance for the different membranes. Taking 95 °C as a reference point, the change in water partial pressure by operating at 95 °C is predicted to reduce membrane area by 84.7% relative to operation at 50 °C. However, the reduction in area for the NaA operating at 95 °C is predicted to be only 80.5%, which is the result of declining water permeance with increasing temperature. Conversely, the area reduction achieved by operating the PVA 4100 and 4101 membranes at 95 °C relative to 50 °C, would be 92.3% and 94.5%, respectively — a noticeably greater reduction than that due simply to the improved driving force. The PVA 4100 and 4101 have a stated maximum continuous operating temperature of 100 °C, whereas the maximum operating temperature for NaA membranes is higher, generally 120 °C and above. As a result, although NaA membranes become less efficient at higher temperatures, this is more than counteracted in PV separations by the natural increase in vapor pressure driving force.

Additional process calculations were carried out to probe the effect of variable permeances on single-pass PV system calculations that incorporate non-zero permeate pressures and nonisothermal operation. The latter involves specifying a maximum allowable temperature drop before interstage reheating is needed. The calculated effect of variable permeances on nonisothermal operations depended on the type of membrane. For example, allowing a 10 °C temperature drop before interstage reheating for the baseline separation scenario increased the amount of NaA, PVA 4100, and PVA4101 membrane area by 15.8,26.7, and 30.9%, respectively, when temperature-dependent permeances were incorporated, compared to an increase of 18.5% with temperature-independent permeances (fixed at the 95 °C values). Thus, most of the increased area was due to the declining driving force as the feed-side temperature was allowed to fall as low as 85 °C. However, there was a noticeable increase in area for the PVA membranes as they became less efficient with the falling temperature. The NaA membrane became slightly more efficient and so the increase in area was smaller than that seen without including temperature-dependent permeances. The effect of temperature-dependent permeances on ethanol recovery with the NaA membrane for the 10 °C temperature drop example was quite small — less than 1%. Thus, incorporating a feed-side temperature drop in the process calculation results in a noticeable change in the predicted amount of membrane area required for all the membranes, but the magnitude of the change and the difference between the areas calculated with the temperature-independent and temperature-dependent permeances depended on the membrane type.

The effect of variable permeances on the change in membrane area due to non-zero permeate pressures was similarly small. For example, increasing permeate pressure from 0 to 4 kPa is predicted to increase the amount of NaA membrane area required by 59.5% when permeances are fixed at 10 wt% water values. With variable permeances, the area increase was only slightly more: 65.9%. The effect of this same increase in permeate pressure on ethanol losses to the permeate was a 62% increase when permeances were fixed at 10 wt% water values, and this was about the same as the corresponding area increase; however, the ethanol loss increased about twice that much (120%) when variable permeances were used. Most of the increased area is needed at the lowest feed-side water concentrations where the water partial pressure driving force is most impacted by the permeate pressure increase. Under these same conditions, the ethanol permeance for the NaA membrane is the highest. This combination leads to a relative increase in ethanol transfer to the permeate when the variable permeances are used for the NaA membrane, compared to the permeances fixed at 10 wt% water values. Thus, as might be expected, variable permeance expressions are likely to have a noticeable impact when the permeances respond strongly to composition or temperature.

## CONCLUSIONS

The permeability of most PV/V⋅P membrane materials is affected by the composition of the feed fluid and the process temperature. The multiparameter permeability expression used in this work (Eqn (4)) was able to represent the effect of changes in feed-side composition and temperature on the water and ethanol permeances of a NaA membrane and two PVA membranes based on PV data reported for them in the literature. These examples demonstrated how different types of membranes (NaA vs. PVA) and different preparations of the same type of membrane (degree of PVA crosslinking) required different forms of the permeability expression and resulted in noticeably different magnitudes and signs of the coefficients.

The permeability expression enabled evaluation of how PV/V⋅P process performance estimates would be affected if concentration- and temperature-dependent permeabilities were incorporated, compared to assuming constant permeabilities. For the NaA and PVA membranes, the effect of changing composition and temperature on the PV water partial pressure driving force was more significant than on the water permeances, meaning that the largest portion of the changes observed in estimated membrane area were due to driving force changes. Nevertheless, replacing constant permeances in the PV single-pass process calculator with the multiparameter permeability expressions resulted in significant changes to the estimated amount of membrane area required to reduce water content from 10 to 1 wt% and to the estimated amount of ethanol transferred to the permeate stream. It is expected that these observations will be true for many other types of membranes used in PV and V⋅P applications. Thus, unless the PV/V⋅P process being modeled is expected to operate at a constant temperature and in a narrow concentration range, process performance estimates would be significantly improved by inclusion of concentration- and temperature-dependent permeabilities or permeances.

Although the permeability expression employed in this work was adaptable and applicable to the example data sets, care should be taken in extrapolating outside the concentration or temperature ranges of the experimental data with this, or any, expression that has been fit to experimental results. Ideally, experimental performance data that covers the range of water concentrations and temperatures expected in the process would be available. Refinements to the permeability expression, such as adding a composition-dependent Arrhenius parameter or the average activity in the selective layer, might prove useful for certain types of membranes or solvent/water mixtures. However, each added flexibility in the expression requires a more extensive data set to populate the coefficients. Aside from expanded permeability expressions, the process calculators could be enhanced in the future by accounting for the feed-side boundary layer mass transfer resistance by incorporating a generic boundary layer mass transfer expression with user-defined coefficients.

## Supplementary Material

Supplementary Info 1

Supplementary Info 2

## Figures and Tables

**Table 1.** Constants for use in the permeability expression (Eqn (4)) for the NaA and PVA membrane examples for ethanol/water pervaporation treatment

Membrane	$P_{ow}\left(\text{kmol}\;\text{m}{\text{m}}^{-2}{\text{s}}^{-1}\text{kP}{\text{a}}^{-1}\right)$ ^c^	$P_{os}\left(\text{kmol}\;\text{m}{\text{m}}^{-2}{\text{s}}^{-1}\text{kP}a^{-1}\right)$ ^c^	Default Thick-ness, ℓ(μm)	$T_{0}\left(\text{K}\right)$	$E_{w}(\text{kJ}\;\text{kmo}{\text{l}}^{-1})$	$A_{w}$	$B_{w}$	$C_{w}$	$D_{w}$	$F_{w}$	$G_{w}$	$H_{w}$	$E_{s}\left(\text{kJ}\;\text{kmo}{\text{l}}^{-1}\right)$	$A_{s}$	$B_{s}$	$C_{s}$	$D_{s}$	$F_{s}$	$G_{s}$	$H_{s}$
NaA^a^	1.3090 × 10^−12^	5.3787 × 10^−15^	1	323.15	−5330.09	1	2.3753	−2.1212	0	0	0	0	−6250.65	1	0	0	0	0	−10.46	0
PVA 4100^b^	3.5485 × 10^−13^	1.0799 × 10^−16^	1	368.15	15 208.6	1	0	0	0	0	1.5771	0	15 208.6	1	0	0	0	0	3.5393	0
PVA 4101^b^	2.6355 × 10^−13^	4.0320 × 10^−17^	1	368.15	22 530.1	1	0	0	0	0	1.3796	0	22 530.1	1	0	0	0	0	2.1748	0

Parameters for NaA zeolite membrane calculated from data presented by Kondo *et al*.^11^ Temperature range: 50 to 120 °C. Water concentration range: 1 to 10 wt%.

Parameters for poly(vinyl alcohol) (PVA) membranes PERVAP 4100 and PERVAP 4101 calculated from data presented by Yave.^6^ Temperature range: 60 to 105 °C (Solvent temperature constant, *Es*, assumed to be the same as the water temperature constant). Water concentration range: 0.8 to 15 wt%.

The permeance, in GPU, of a membrane with a default thickness of 1μm is 2.99E15 times the $\text{kmol}\;\text{m}\;{\text{m}}^{-2}s^{-1}\text{kP}a^{-1}$ permeability value. Thus, the NaA membrane with $P_{ow}=1.3090\times 10^{-12}\text{kmol}\;\text{m}{\text{m}}^{-2}s^{-1}\text{kP}a^{-1}$ and default thickness of 1μm has a water permeance of 3914 GPU (with aw=0, and T=To).

**Table 2.** Performance characteristics of the example membranes calculated from the respective permeance equations under the feed (10 wt% water) and retentate (1 wt% water) conditions of the 95 °C baseline ethanol/water process scenario

Membrane	$\text{Feed}\;\Pi_{w}\left(\text{GPU}\right)$	$\text{Feed}\;\Pi_{e}\left(\text{GPU}\right)$	Feedα	$\text{Retentate}\;\Pi_{w}\left(\text{GPU}\right)$	$\text{Retentate}\;\Pi_{e}\left(\text{GPU}\right)$	Retentateα
NaA	5016	0.1245	40 280	3488	6.432	542.3
PVA 4100	2115	1.519	1393	1167	0.3999	2919
PVA 4101	1441	0.3122	4616	856.5	0.1375	6230

## NOMENCLATURE

**ROMAN SYMBOLS**
$a_{i}\;$: activity of compound i
A: membrane area, $m^{2}$
$A_{i}\;$: parameter in permeability expression for compound i
$A_{k}\;$: sub-unit membrane area, $m^{2}$
$B_{i}\;$: parameter in permeability expression for compound i
$C_{i}\;$: parameter in permeability expression for compound i
$D_{i}\;$: parameter in permeability expression for compound i
$E_{i}\;$: Arrhenius-type parameter in permeability expression for compound $i,\;kJ{kmol}^{-1}$
$F_{i}\;$: parameter in permeability expression for compound i
$G_{i}\;$: parameter in permeability expression for compound i
$H_{i}\;$: parameter in permeability expression for compound i
$j_{i}\;$: molar flux of compound i through the membrane, $\text{kmol}m^{-2}{\;s}^{-1}$
$J_{i}\;$: mass flux of compound i through the membrane, $\text{kg}m^{-2}{\;h}^{-1}$
l: thickness of membrane selective layer, m
$M_{i}\;$: molecular weight of compound $i,\;\text{kg}{kmol}^{-1}$
$\Delta N_{i}\;$: amount of compound i transferred across the membrane in a batch sub-unit, kmol
$\Delta {\dot{N}}_{i}\;$: rate of transfer of compound i across the membrane in a single-pass sub-unit, $\text{kmol}s^{-1}$
$p_{i}^{\text{sat }}\;$: saturated vapor pressure of compound i,kPa
$p_{i}^{F}\;$: partial pressure of compound i in the feed-side vapor or feed-side liquid, kPa
$p_{i}^{F^{*}}\;$: hypothetical feed-side partial vapor pressure of compound i,kPa
$p_{i}^{p}\;$: partial pressure of compound i in the permeate vapor, kPa
$p_{\text{Total }}^{F}\;$: total feed-side pressure, kPa
$p_{\text{Total }}^{P}\;$: total permeate pressure, kPa
$P_{i}\;$: permeability of i in membrane selective layer, $\text{kmol}\;m\;m^{-2}{\;s}^{-1}{kPa}^{-1}$
$P_{oi}\;$: pre-exponential in permeability expression for compound $i,\text{kmol}\;m\;m^{-2}{\;s}^{-1}{kPa}^{-1}$
R: gas constant, $8.314\;kJ{kmol}^{-1}{\;K}^{-1}$
$\Delta t_{k}\;$: time interval for a batch sub-unit, $s^{-1}$
T: temperature, K
$T_{0}\;$: reference temperature, K
$x_{i}\;$: mole fraction of component i in the feed-side vapor or feed-side liquid
$y_{i}\;$: mole fraction of component i in the permeate vapor

## GREEK SYMBOLS

$\alpha_{ij}\;$: molar permselectivity (‘selectivity’) for compound i relative to compound j
$\gamma_{i}\;$: activity coefficient of compound i
$\Pi_{i}\;$: permeance of compound i through membrane, GPU or $\text{kmol}m^{-2}{\;s}^{-1}{kPa}^{-1}$
