# Supplementary material for: Effect of membrane performance variability with temperature and feed composition on pervaporation and vapor permeation system design for solvent drying
Source: J Chem Technol Biotechnol. Author manuscript; Available in PMC 2023 Oct 12. (PMC10569128; doi:10.1002/jctb.7161)
Supplement: Supplementary Info 1 [file NIHMS1932213-supplement-Supplementary_Info_1.docx]

**Supporting Information**

**“Effect of membrane performance variability with temperature and feed composition on pervaporation and vapor permeation system design for solvent drying”** by Leland M. Vane

**Contents**

[**1.** **Parameters for chemical/physical properties** S2](#_Toc105925608)

[Table S1. Non-Random Two-Liquid (NRTL) thermodynamic model parameters for binary water-solvent mixtures S2](#_Toc105925609)

[Table S2. Antoine saturated vapor pressure parameters S3](#_Toc105925610)

[Table S3. Liquid heat capacity (C_p_) parameters for pure compounds S4](#_Toc105925611)

[Table S4. Enthalpy of vaporization parameters S5](#_Toc105925612)

[**2.** **Activity values for ethanol/water liquid mixtures** S6](#_Toc105925613)

[**3.** **Calculations for NaA zeolite membranes from Kondo et al. (1997)** S7](#_Toc105925614)

[Table S5. Data and calculated performance parameters for **NaA zeolite** membrane. Pervaporation with ethanol/water mixtures and permeate pressure of 0.133 kPa.^7^ S10](#_Toc105925615)

[**4.** **Calculations for PVA-based membranes from Yave et al. (2019)** S11](#_Toc105925616)

[Table S6. Data and calculated performance parameters for Deltamem PERVAP membranes 4100 and 4101. Pervaporation with 10 wt% water in ethanol at 95 °C and permeate pressure of 10 mbar.^8^ S12](#_Toc105925617)

[Table S7. Data and calculated performance parameters for Deltamem **PERVAP 4100** membrane. Pervaporation with ethanol/water at 95 °C and permeate pressure of 10 mbar (1 kPa).^8^ S16](#_Toc105925618)

[Table S8. Data and calculated performance parameters for Deltamem **PERVAP 4101** membrane. Pervaporation with ethanol/water at 95 °C and permeate pressure of 10 mbar (1 kPa).^8^ S17](#_Toc105925619)

[**5.** **References for Supporting Information** S18](#_Toc105925620)

# **Parameters for chemical/physical properties**

## Table S1. Non-Random Two-Liquid (NRTL) thermodynamic model parameters for binary water-solvent mixtures

| **Solvent (s)** | **B_ws_ (K)** | **B_sw_ (K)** | **α** |
| --- | --- | --- | --- |
| Acetone | 653.885 | 377.577 | 0.5856 |
| Acetonitrile | 612.822 | 553.593 | 0.5654 |
| 1-Butanol | 1468.34 | 215.427 | 0.3634 |
| DMAC | 171.435 | −31.6037 | 2.3267 |
| DMF | 470.31 | −267.677 | 0.2768 |
| Ethanol | 670.441 | −55.1681 | 0.3031 |
| Methanol | 307.166 | −24.4933 | 0.3001 |
| MIBK | 1549.38 | 562.189 | 0.3686 |
| MtBE | 1108.82 | 682.05 | 0.3 |
| NMP | 180.083 | −89.2686 | 0.2967 |
| 2-Propanol | 832.981 | 20.0554 | 0.3255 |
| THF | 953.251 | 449.411 | 0.4306 |

From ChemCAD v.8.1 (Chemstations Inc., Houston, Texas, USA).

NRTL parameters for water-solvent mixtures are available from a variety of published and membership- or fee-based sources, including the DECHEMA Chemistry Data Series,^1^ the Design Institute for Physical Properties (DIPPR) database,^2^ or chemical process simulation software programs (e.g., ChemCAD or Aspen Plus).

For a binary system:

$$\ln\gamma_{w}={x_{s}}^{2}\left[ \tau_{sw}\left( \frac{G_{sw}}{x_{w}+x_{s}G_{sw}} \right)^{2}+\frac{\tau_{ws}G_{ws}}{\left( x_{s}+x_{w}G_{ws} \right)^{2}} \right]$$

$$\ln\gamma_{s}={x_{w}}^{2}\left[ \tau_{ws}\left( \frac{G_{ws}}{x_{s}+x_{w}G_{ws}} \right)^{2}+\frac{\tau_{sw}G_{sw}}{\left( x_{w}+x_{s}G_{sw} \right)^{2}} \right]$$

Where:

$$\tau_{ji}=A_{ji}+\frac{B_{ji}}{T}+C_{ji} \ln(T)+D_{ji} T$$

For calculations here, the $A_{ji}$, $C_{ji}$, and $D_{ji}$ parameters were zero.

$$G_{ji}=exp(-\alpha_{ji} \tau_{ji})$$

$$\alpha_{ij}{=\alpha}_{ji}$$

*T* is temperature in Kelvin

## Table S2. Antoine saturated vapor pressure parameters

| **Compound** | ***A*** | ***B*** | ***C*** | ***Reference*** |
| --- | --- | --- | --- | --- |
| Water | 7.074227159 | 1657.441461 | −46.13 | a |
| Acetone | 6.356468668 | 1277.025552 | −35.93 | b |
| Acetonitrile | 6.46100335 | 1462.052373 | −26.651 | a |
| 1-Butanol | 6.382396049 | 1260.756881 | −102.91 | a |
| DMAC | 5.694907884 | 1253.330445 | −100.32 | a |
| DMF | 5.973724942 | 1367.028741 | −81.342 | a |
| Ethanol | 7.337409615 | 1652.056209 | −41.68 | a |
| Methanol | 7.163691822 | 1560.593791 | −35.225 | a |
| MIBK | 5.949404451 | 1256.717942 | −70.75 | a |
| MtBE | 6.254887189 | 1265.403832 | −30.63 | b |
| NMP | 6.673198737 | 1979.68 | −50.95 | c |
| 2-Propanol | 6.79627869 | 1350.351833 | −73.546 | a |
| THF | 6.120082182 | 1202.300844 | −46.9 | a |

Saturated Vapor Pressure (kPa)

$$p^{sat}= {10}^{\left( A-\frac{B}{T+C} \right)}$$

Where *T* is in Kelvin

References:

1. ChemCAD v.8.1
2. Towler G and Sinnott RK, Appendix C: Physical Property Data Bank, in Chemical Engineering Design - Principles, Practice and Economics of Plant and Process Design (2nd Edition). Elsevier (2013).^3^
3. Dortmund database: http://ddbonline.ddbst.com/AntoineCalculation/AntoineCalculationCGI.exe^4^

In addition to these references, Antoine parameters can be obtained from a variety of published and membership- or fee-based sources, including the DECHEMA Chemistry Data Series,^1^ the NIST chemistry webbook,^5^ the Design Institute for Physical Properties (DIPPR) database,^2^ or chemical process simulation software programs (e.g., ChemCAD or Aspen Plus).

## Table S3. Liquid heat capacity (C_p_) parameters for pure compounds

|  | **Liquid heat capacity parameters (kJ kmol^−1^ K^−1^)** | | | | | |
| --- | --- | --- | --- | --- | --- | --- |
| **Compound** | **A** | **B** | **C** | **D** | **E** | Cp at normal boiling point |
| Water | −22.4167 | 0.876959 | −0.00257 | 2.48E−06 | 0 | 75.97 |
| Acetone | 354.7694 | −3.51085 | 0.01873 | −4.3E−05 | 3.79E−08 | 132.41 |
| Acetonitrile | −1110.88 | 16.36478 | −0.08336 | 0.000187 | −1.6E−07 | 102.70 |
| 1-Butanol | −368.4 | 5.816157 | −0.02334 | 3.78E−05 | −1.5E−08 | 237.23 |
| DMAC | 51.543 | 0.89035 | −0.002264 | 2.358E−06 | 0 | 205.54 |
| DMF | 59.52702 | 0.60708 | −0.00162 | 1.86E−06 | 0 | 168.10 |
| Ethanol | 238.313 | −2.38069 | 0.013317 | −3.2E−05 | 3.15E−08 | 138.28 |
| Methanol | 428.8995 | −5.33154 | 0.028463 | −6.5E−05 | 5.49E−08 | 88.97 |
| MIBK | 128.9726 | 0.528062 | −0.0015 | 2.24E−06 | 0 | 238.70 |
| MtBE | 529.9685 | −6.04474 | 0.034294 | −8.1E−05 | 7.04E−08 | 202.75 |
| NMP | 29.425 | 0.42736 | 0 | 0 | 0 | 232.51 |
| 2-Propanol | 873.1334 | −10.0591 | 0.045744 | −8.4E−05 | 5.56E−08 | 202.52 |
| THF | 63.39307 | 0.40257 | −0.00127 | 1.83E−06 | 0 | 125.10 |

$$\boldsymbol{C}_{\boldsymbol{p}} \left( \text{kJ kmol}\text{-1}\text{ K}\text{-1} \right)\boldsymbol{=A+BT+C}\boldsymbol{T}^{\boldsymbol{2}}\boldsymbol{+D}\boldsymbol{T}^{\boldsymbol{3}}\boldsymbol{+E}\boldsymbol{T}^{\boldsymbol{4}}$$

*T* in Kelvin.

Reference: The parameters for all compounds except NMP are from Yaws' Critical Property Data for Chemical Engineers and Chemists^6^. NMP values from ChemCAD v8.1.

In addition to these references, heat capacity parameters can be obtained from a variety of published and membership- or fee-based sources, including the DECHEMA Chemistry Data Series,^1^ the NIST chemistry webbook,^5^ the Design Institute for Physical Properties (DIPPR) database,^2^ or chemical process simulation software programs (e.g., ChemCAD or Aspen Plus). For a constant heat capacity, that value can be entered as “A” and zeroes entered for all other parameters.

## Table S4. Enthalpy of vaporization parameters

| **Compound** | ***A*** | ***B*** | ***n*** | $\Delta H_{i}^{vap}$at normal boiling point (kJ kmol^−1^) |
| --- | --- | --- | --- | --- |
| Water | 54000 | 647.13 | 0.34 | 40316.34 |
| Acetone | 49244.1 | 508.2 | 0.481 | 29794.79 |
| Acetonitrile | 43081.9 | 545.5 | 0.335 | 30298.72 |
| 1-Butanol | 63024.2 | 562.93 | 0.318 | 43233.97 |
| DMAC | 61355.9 | 658 | 0.374 | 40642.50 |
| DMF | 59354.5 | 647 | 0.381 | 39477.33 |
| Ethanol | 60803.6 | 516.25 | 0.38 | 39390.01 |
| Methanol | 52722.7 | 512.58 | 0.377 | 35139.05 |
| MIBK | 57680 | 571.4 | 0.416 | 35775.18 |
| MtBE | 45027.6 | 497.1 | 0.434 | 28173.84 |
| NMP | 63749.4 | 724 | 0.332 | 44716.23 |
| 2-Propanol | 58982.4 | 508.31 | 0.326 | 39869.55 |
| THF | 44438.7 | 540.15 | 0.391 | 30259.84 |

**Enthalpy of Vaporization (kJ kmol^−1^) =**

$$\Delta H_{i}^{vap}=A\left( 1-\frac{T}{B} \right)^{n}$$

Where $T$ is in Kelvin

Reference: Yaws' Critical Property Data for Chemical Engineers and Chemists^6^

In addition to these references, enthalpy of vaporization parameters can be obtained from a variety of published and membership- or fee-based sources, including the DECHEMA Chemistry Data Series,^1^ the NIST chemistry webbook,^5^ the Design Institute for Physical Properties (DIPPR) database,^2^ or chemical process simulation software programs (e.g., ChemCAD or Aspen Plus). For a constant enthalpy of vaporization, that value can be entered as “A”, any non-zero value entered for “B”, and a zero entered for “n”.

# **Activity values for ethanol/water liquid mixtures**

The activity ($a_{i}$) of water and solvent in the feed-side liquid of a pervaporation (PV) process were calculated according to Equation 8 in the main paper ($a_{i}\text{(PV)}=x_{i} \gamma_{i}$) where $x_{i}$ is the mole fraction and $\gamma_{i}$ is the activity coefficient of compound *i* in the feed-side liquid, with $\gamma_{i}$ calculated herein based on the NRTL thermodynamic model with parameters given in Table S1 using the “VLE” calculator in the Excel file “Solvent Dehydration Calculators-v09Jun2022.xlsm.” The activities of ethanol and water calculated in this manner for a water concentration range of 0 to 20 wt% and a liquid temperature of 95 °C are shown in Figure S1 along with the corresponding mole fractions of the compounds to illustrate the effect of water concentration on activity.

As indicated in the figure, ethanol activity ($a_{e}$) in this concentration range is very similar to the ethanol mole fraction due to $\gamma_{e}$ being close to unity. However, $\gamma_{w}$ ranges from 2.46 to 1.65 as water content increases from 0 to 20 wt% resulting in values of water activity ($a_{w}$) that are noticeably larger than the water mole fraction, particularly at low concentrations.

Figure S1. Activities and mole fractions of ethanol and water in a water/ethanol liquid mixture as a function of the water content (in weight %) at 95 °C calculated using the NRTL parameters given in Table S1.

# **Calculations for NaA zeolite membranes from Kondo et al. (1997)**

Kondo et al. provides bench-scale PV performance data for NaA zeolite membranes prepared by researchers at Mitsui Engineering and Shipbuilding, which has commercialized NaA membranes.^7^ The effective membrane area was 289 cm^2^. In Table 2 of the reference, data for a NaA selective layer grown on a support with a composition of 65:35 (wt%:wt%) Al_2_O_3_:SiO_2_ were presented and will be used herein. Data was obtained at water concentrations ranging from 0.05 wt% to 10.09 wt% and temperatures ranging from 50 to 120 °C. Permeate pressure was stated to be 0.133 kPa. Using the “Flux Converter” calculator in the Excel file “Solvent Dehydration Calculators-v09Jun2022.xlsm”, those raw data were converted to permeances and selectivity values. Because of the very low driving force associated with the 0.05 wt% water data points, which lead to a negative water permeance at 50 °C, data obtained with 0.05 wt% water in the liquid were not considered to be reliable for the purposes of this work. The values from Table 2 of the reference used in this work are presented in Table S5 along with the calculated performance values.

Using the three-dimensional (3-D) curve-fitting function of SigmaPlot software (v.14, Systat Software, Inc.), all of the data in Table S5 were used to determine the coefficients in the permeability equations for water and ethanol. The graphs showing the 3d data plot and model fits for water permeance and ethanol permeance are shown in Figures S2 and S3, respectively. The curve fit equations for the permeances are:

$$\Pi_{w}\text{(GPU)}=3914.0\text{ }\exp\left[ \left( \frac{-5330.09}{8.314} \right)\left( \frac{1}{323.15\text{K}}-\frac{1}{T} \right) \right]\left( 1+2.375306a_{w}-2.1212307{a_{w}}^{2} \right)$$

$$\Pi_{e}\text{(GPU)}=16.082 \exp\left[ \left( \frac{-6250.65}{8.314} \right)\left( \frac{1}{323.15\text{K}}-\frac{1}{T} \right) \right]\exp(-10.46a_{w})$$

Using a default membrane thickness of 1 μm and converting to SI units results in the following expressions for permeability:

$$P_{w}\text{(}\text{kmol}\text{-m/m2-s-kPa}\text{) = }1.30903\text{E-12 }\exp\left[ \left( \frac{-5330.09}{8.314} \right)\left( \frac{1}{323.15\text{K}}-\frac{1}{T} \right) \right]\times\left( 1+2.375306a_{w}-2.1212307{a_{w}}^{2} \right)$$

$$P_{e}\text{(}\text{kmol}\text{-m/m2-s-kPa}\text{)}=5.378729\text{E-15} \exp\left[ \left( \frac{-6250.65}{8.314} \right)\left( \frac{1}{323.15\text{K}}-\frac{1}{T} \right) \right]\exp(-10.46a_{w})$$

Note that only the pre-exponential parameter needs to be changed when converting between GPU and SI units or between permeance and permeability. These expressions were used to represent the performance of the NaA membrane in the present manuscript. As noted in the manuscript, these relationships are not meant to imply that every membrane of this type will have the same performance, rather they are used to make observations of how adding variable permeabilities to the dehydration process calculations can affect predictions about the performance of such processes.

Note that the format of the equations for water and ethanol are different. Water permeance is represented by a quadratic expression with water activity, while ethanol permeance is represented by an exponential decay relationship with water activity. As with any curve fit relationship, caution should be taken in extrapolating beyond the range of water concentrations (1-10 wt%) and temperatures (50-120 °C) of the data set.


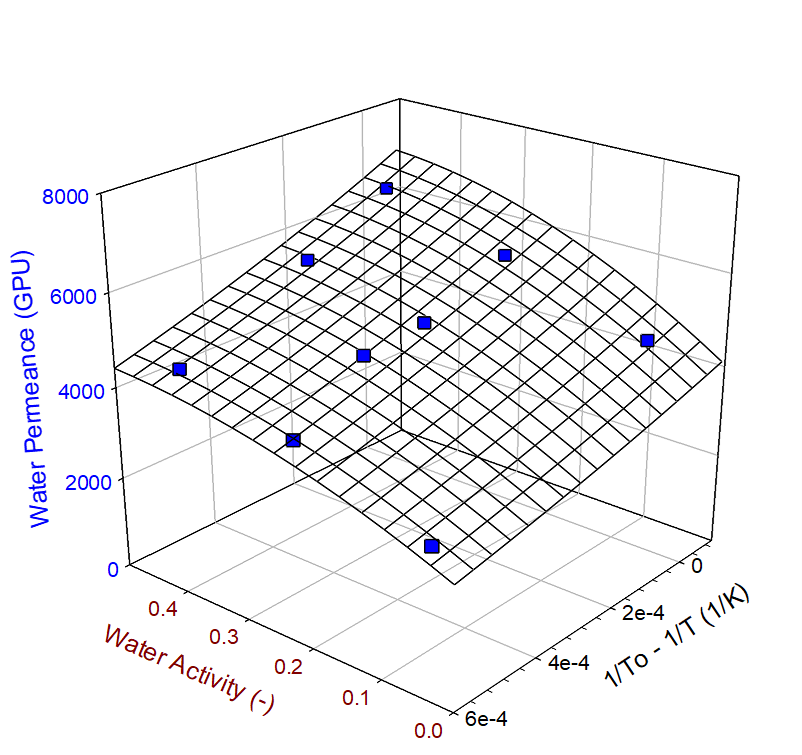


Figure S2. 3-D curve fit used to generate coefficients for NaA water permeance equation. The water activities represent three separate water concentrations: 1.0, 5.02, and 10.09 wt%.


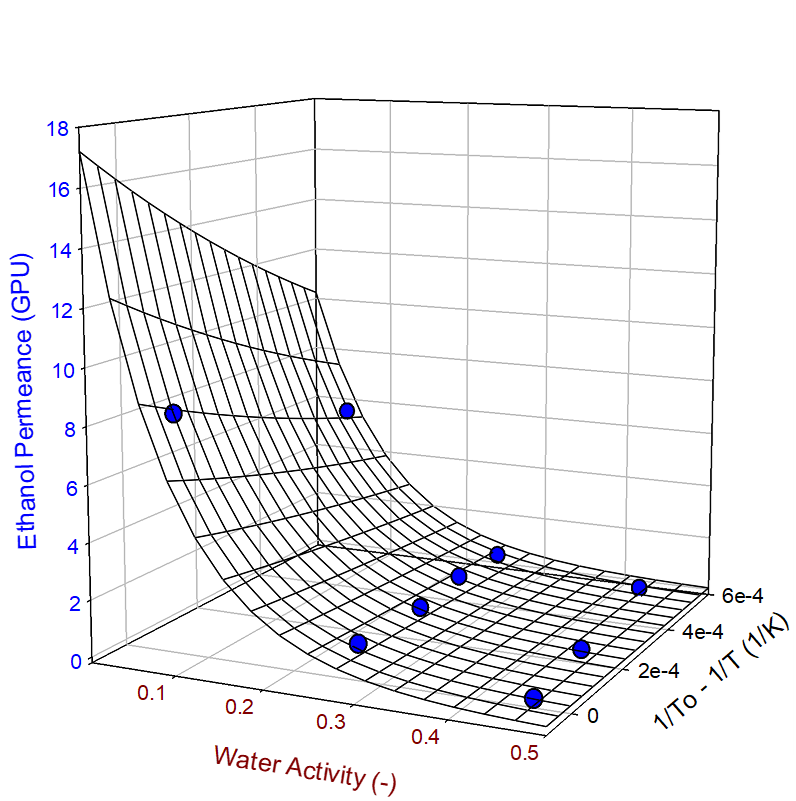


Figure S3. 3-D curve fit used to generate coefficients for NaA ethanol permeance equation.

## Table S5. Data and calculated performance parameters for **NaA zeolite** membrane. Pervaporation with ethanol/water mixtures and permeate pressure of 0.133 kPa.^7^

| Feed Temp. (°C) | Feed water conc.* (wt frx) | Total Flux*  (kg/m2-h) | Separation Factor* | Water Partial P (kPa) | Ethanol Partial P (kPa) | Water Activity  (-) | EtOH Activity  (-) | Water Permeance (kmol/m2-s-kPa) | EtOH Permeance (kmol/m2-s-kPa) | Selectivity | Water Permeance (GPU) | EtOH Permeance (GPU) |
| --- | --- | --- | --- | --- | --- | --- | --- | --- | --- | --- | --- | --- |
| 50 | 0.01 | 0.079 | 500 | 0.7745 | 28.6463 | 0.06279 | 0.97509 | 1.562E−06 | 2.749E−09 | 568.1 | 4669.6 | 8.2198 |
| 50 | 0.0502 | 0.396 | 4800 | 3.3627 | 26.0631 | 0.27263 | 0.88716 | 1.883E−06 | 3.597E−10 | 5235.1 | 5630.2 | 1.0755 |
| 50 | 0.1009 | 0.772 | 46000 | 5.6885 | 23.4821 | 0.46119 | 0.79931 | 2.142E−06 | 3.839E−11 | 55800.6 | 6405.4 | 0.1148 |
| 75 | 0.0502 | 1.1 | 5900 | 10.2701 | 78.5101 | 0.26620 | 0.88739 | 1.668E−06 | 2.700E−10 | 6176.1 | 4986.7 | 0.8074 |
| 75 | 0.1009 | 2.08 | 42000 | 17.3337 | 70.7675 | 0.44928 | 0.79987 | 1.864E−06 | 3.759E−11 | 49591.1 | 5573.9 | 0.1124 |
| 95 | 0.0502 | 2.35 | 5100 | 22.0791 | 167.9666 | 0.26108 | 0.88751 | 1.645E−06 | 3.118E−10 | 5275.8 | 4918.5 | 0.9323 |
| 120 | 0.01 | 0.848 | 520 | 11.7337 | 422.7155 | 0.05908 | 0.97512 | 9.461E−07 | 1.935E−09 | 489.1 | 2828.9 | 5.7843 |
| 120 | 0.0502 | 4.3 | 5600 | 50.6163 | 384.7845 | 0.25485 | 0.88762 | 1.309E−06 | 2.269E−10 | 5769.1 | 3913.7 | 0.6784 |
| 120 | 0.1009 | 8.37 | 47000 | 85.2783 | 346.9558 | 0.42936 | 0.80036 | 1.515E−06 | 2.757E−11 | 54964.0 | 4531.2 | 0.0824 |

*Values from Table 2 in Kondo et al.^7^

# **Calculations for PVA-based membranes from Yave et al. (2019)**

Yave et al. provides performance data for two commercialized polyvinyl alcohol (PVA) membranes from Deltamem.^8^ The PERVAP 4100 and PERVAP 4101 membranes consist of thin selective layers of crosslinked PVA on a microporous support layer. The PVA in the 4101 has a higher degree of crosslinking than the PVA of the 4100. As a result, the 4101 swells less than the 4100 at the same water activity and the permeances of the 4101 will change less with water activity than for the 4100.^8^

The reference provides a graph (Fig. 2 in the reference) showing the effect of feed water concentration on water flux and ethanol flux at a feed temperature of 95 °C and permeate pressure of 10 mbar. In a private communication, the author of that article provided the data points for that graph. Using the “Flux Converter” calculator in the Excel file “Solvent Dehydration Calculators-v09Jun2022.xlsm”, those raw data were converted to permeances and selectivity values. The raw data and the calculated performance properties are listed in Table S7 and Table S8 below.

In addition, the reference provides information regarding the effect of temperature on the water permeance (but not ethanol permeance).

The following correlations were developed for the **PERVAP 4100** from the data at 95 °C:

$$\Pi_{w}\text{(GPU)}=1061.01166 \exp\left( 1.5771484a_{w} \right) \exp\left[ \left( \frac{E_{w}}{R} \right)\left( \frac{1}{368.15\text{K}}-\frac{1}{T} \right) \right]$$

$$\Pi_{e}\text{(GPU)}=0.32289061 \exp(3.5393051a_{w}) \exp\left[ \left( \frac{E_{e}}{R} \right)\left( \frac{1}{368.15\text{K}}-\frac{1}{T} \right) \right]$$

Or, in SI units:

$$\Pi_{w}\text{(kmol/m2-s-kPa)}=3.54853\text{E-7} \exp(1.5771484a_{w}) \exp\left[ \left( \frac{E_{w}}{R} \right)\left( \frac{1}{368.15\text{K}}-\frac{1}{T} \right) \right]$$

$$\Pi_{e}\text{(kmol/m2-s-kPa)}=1.07990\text{E-10} \exp(3.5393051a_{w}) \exp\left[ \left( \frac{E_{e}}{R} \right)\left( \frac{1}{368.15\text{K}}-\frac{1}{T} \right) \right]$$

Where $T$ is in Kelvin and 368.15 K is the reference temperature of 95 °C for the PVA membrane data set. $E_{w}$ and $E_{e}$ are the exponential parameters in the Arrhenius-type term for water and ethanol permeance, respectively, for the specific membrane.

The following correlations were developed for the **PERVAP 4101** from the data at 95 °C:

$$\Pi_{w}\text{(GPU)}=788.0245 \exp(1.3795548a_{w})\exp\left[ \left( \frac{E_{w}}{R} \right)\left( \frac{1}{368.15\text{K}}-\frac{1}{T} \right) \right]$$

$$\Pi_{e}\text{(GPU)}=0.120558 \exp(2.1748373a_{w})\exp\left[ \left( \frac{E_{e}}{R} \right)\left( \frac{1}{368.15\text{K}}-\frac{1}{T} \right) \right]$$

Or, in SI units:

$$\Pi_{w}\text{(kmol/m2-s-kPa)}=2.63553\text{E-7} \exp(1.3795548a_{w})\exp\left[ \left( \frac{E_{w}}{R} \right)\left( \frac{1}{368.15\text{K}}-\frac{1}{T} \right) \right]$$

$$\Pi_{e}\text{(kmol/m2-s-kPa)}=4.03203\text{E-11} \exp(2.1748373a_{w})\exp\left[ \left( \frac{E_{e}}{R} \right)\left( \frac{1}{368.15\text{K}}-\frac{1}{T} \right) \right]$$

Using the water flux at two temperatures for a 10 wt% water feed from Yave et al., the exponential parameter in the Arrhenius-type term for the water permeance can be estimated.

## Table S6. Data and calculated performance parameters for Deltamem PERVAP membranes 4100 and 4101. Pervaporation with 10 wt% water in ethanol at 95 °C and permeate pressure of 10 mbar.^8^

| Membrane | Temperature (°C) | Water Flux (kg/m^2^-h) | Water Partial P (kPa) | Water activity  (-) | Water Permeance (kmol/m^2^-s-kPa) | Water Permeance (GPU) | Calc. Water Permeance at 95 °C (GPU) |
| --- | --- | --- | --- | --- | --- | --- | --- |
| 4100 | 60 | 0.202 | 9.0425 | 0.45364 | 3.8719E−07 | 1157.7 | 2169.9 |
| 4100 | 105 | 2.400 | 52.346 | 0.43316 | 7.2070E−07 | 2154.9 | 2100.9 |
|  |  |  |  |  |  |  |  |
| 4101 | 60 | 0.120 | 9.0425 | 0.45364 | 2.2997E−07 | 687.6 | 1473.4 |
| 4101 | 105 | 1.960 | 52.346 | 0.43316 | 5.8856E−07 | 1759.8 | 1432.4 |

Note: Water Permeance (GPU) = 2.99E9 * Water Permeance (kmol/m^2^-s-kPa)

From this information, the values of $E_{w}$ for the 4100 and 4101 membranes are calculated to be 15,208.6 and 22,530.1 kJ/kmol, respectively.

For the purposes of this work, the exponential parameter in the Arrhenius-type term for ethanol permeance is assumed to be the same as that of water. This is unlikely to be the actual case, but data was not provided in Yave to calculate it. In addition, the single-pass dehydration area requirements reported in this work will only depend on water permeance because permeate pressure will be assumed negligible so that permeate composition will not be important and ethanol lost from the feed will have a negligible effect on feed water composition.

As indicated in Table S6, the water permeance of the PVA membranes increases with temperature. This is the opposite of the behavior reported for the NaA membrane.

These expressions were used to represent the performance of PVA membranes in the present manuscript. As noted in the manuscript and above for NaA membranes, these relationships are not meant to imply that every membrane of this type will have the same performance, rather they are used to make observations of how adding variable permeabilities to the dehydration process calculations can affect predictions about the performance of such processes.

Note that the format of the permeance equations for water and ethanol are the same. Both water and ethanol are represented by an exponential relationship with water activity. The exponential coefficient for ethanol is larger than that for water, indicating that ethanol permeance increases more rapidly than water permeance as water activity is increased. As with any curve fit relationship, caution should be taken in extrapolating beyond the range of water concentrations (0.8-15.4 wt%) and temperatures (60-105 °C) of the data set. The recommended maximum long term operating temperature for these PVA membranes is 100 °C.^9^


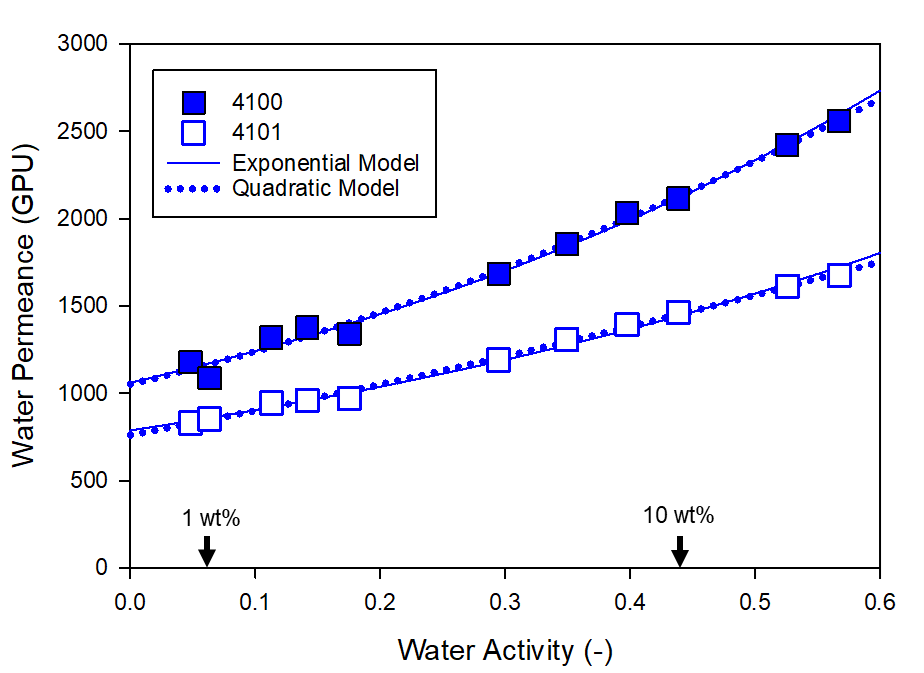


Figure S4. Curve fitting to generate coefficients for PVA membrane water permeance equations. Both quadratic and exponential expressions were fit to the data. The range of water activities of the symbols represents the 0.788 to 15.39 wt% range in water concentration of the data.


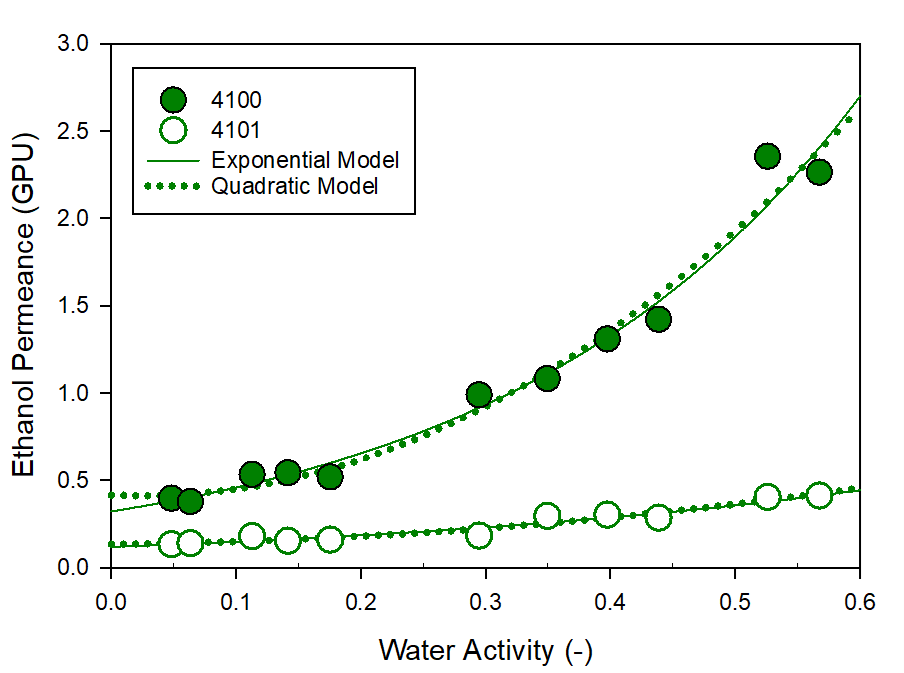


Figure S5. Curve fitting to generate coefficients for PVA membrane ethanol permeance equations. Both quadratic and exponential expressions were fit to the data.

Figure S6. Selectivities for the PVA membranes resulting from the permeance curve fits shown in Figures S4 and S5. The selectivity values for very low water activities tail away from the trend in the data set when the quadratic models were used. The exponential models appear to better represent the selectivity values and, as a result, these models were used in the calculations presented in the manuscript.

## Table S7. Data and calculated performance parameters for Deltamem **PERVAP 4100** membrane. Pervaporation with ethanol/water at 95 °C and permeate pressure of 10 mbar (1 kPa).^8^

| Feed water conc.* (wt frx) | Water Flux*  (g/m2-h) | Ethanol Flux*  (g/m2-h) | Separation Factor | Water Partial P (kPa) | Ethanol Partial P (kPa) | Water Activity  (-) | EtOH Activity  (-) | Water Permeance (kmol/m2-s-kPa) | EtOH Permeance (kmol/m2-s-kPa) | Selectivity | Water Permeance (GPU) | EtOH Permeance (GPU) |
| --- | --- | --- | --- | --- | --- | --- | --- | --- | --- | --- | --- | --- |
| 0.007884 | 78.78 | 4.0969 | 2419.8 | 4.062 | 185.52 | 0.04803 | 0.98027 | 3.9413E−07 | 1.3316E−10 | 2959.7 | 1178.45 | 0.39816 |
| 0.010493 | 103.011 | 3.8755 | 2506.5 | 5.352 | 184.32 | 0.06329 | 0.97392 | 3.6374E−07 | 1.2679E−10 | 2868.9 | 1087.58 | 0.37909 |
| 0.019342 | 244.669 | 5.347 | 2320.0 | 9.538 | 180.37 | 0.11278 | 0.95305 | 4.4144E−07 | 1.7875E−10 | 2469.6 | 1319.91 | 0.53446 |
| 0.024749 | 327.407 | 5.379 | 2398.5 | 11.957 | 178.05 | 0.14139 | 0.94078 | 4.6048E−07 | 1.8216E−10 | 2527.8 | 1376.84 | 0.54467 |
| 0.031456 | 402.08 | 5.058 | 2447.7 | 14.820 | 175.26 | 0.17524 | 0.92604 | 4.4844E−07 | 1.7402E−10 | 2577.0 | 1340.85 | 0.52031 |
| 0.058376 | 875.09 | 9.064 | 1557.3 | 24.932 | 165.00 | 0.29481 | 0.87186 | 5.6371E−07 | 3.3122E−10 | 1701.9 | 1685.50 | 0.99035 |
| 0.072785 | 1149.8 | 9.614 | 1523.6 | 29.547 | 160.08 | 0.34938 | 0.84582 | 6.2098E−07 | 3.6213E−10 | 1714.8 | 1856.72 | 1.08278 |
| 0.086948 | 1437.7 | 11.302 | 1335.8 | 33.617 | 155.57 | 0.39751 | 0.82201 | 6.7959E−07 | 4.3804E−10 | 1551.4 | 2031.97 | 1.30975 |
| 0.10036 | 1656.7 | 11.959 | 1241.8 | 37.091 | 151.59 | 0.43859 | 0.80098 | 7.0774E−07 | 4.7568E−10 | 1487.8 | 2116.14 | 1.42228 |
| 0.13427 | 2285.2 | 18.633 | 790.76 | 44.473 | 142.61 | 0.52588 | 0.75355 | 8.1046E−07 | 7.8779E−10 | 1028.8 | 2423.27 | 2.35549 |
| 0.153925 | 2608.3 | 17.335 | 827.05 | 47.983 | 138.04 | 0.56739 | 0.72937 | 8.5596E−07 | 7.5721E−10 | 1130.4 | 2559.31 | 2.26406 |

*Values provided by W. Yave for Figure 2 in Yave et al.^8^

## Table S8. Data and calculated performance parameters for Deltamem **PERVAP 4101** membrane. Pervaporation with ethanol/water at 95 °C and permeate pressure of 10 mbar (1 kPa).^8^

| Feed water conc.* (wt frx) | Water Flux*  (g/m2-h) | Ethanol Flux*  (g/m2-h) | Separation Factor | Water Partial P (kPa) | Ethanol Partial P (kPa) | Water Activity  (-) | EtOH Activity  (-) | Water Permeance (kmol/m2-s-kPa) | EtOH Permeance (kmol/m2-s-kPa) | Selectivity | Water Permeance (GPU) | EtOH Permeance (GPU) |
| --- | --- | --- | --- | --- | --- | --- | --- | --- | --- | --- | --- | --- |
| 0.007884 | 55.293 | 1.38773 | 5014.0 | 4.062 | 185.52 | 0.04803 | 0.98027 | 2.77546E−07 | 4.510E−11 | 6153.5 | 829.86 | 0.13486 |
| 0.010493 | 80.655 | 1.44507 | 5263.3 | 5.352 | 184.32 | 0.06329 | 0.97392 | 2.85292E−07 | 4.727E−11 | 6035.0 | 853.02 | 0.14135 |
| 0.019342 | 175.13 | 1.8147 | 4893.0 | 9.538 | 180.37 | 0.11278 | 0.95305 | 3.16141E−07 | 6.066E−11 | 5211.4 | 945.26 | 0.18139 |
| 0.024749 | 228.02 | 1.5263 | 5887.0 | 11.957 | 178.05 | 0.14139 | 0.94078 | 3.20809E−07 | 5.169E−11 | 6206.6 | 959.22 | 0.15455 |
| 0.031456 | 291.16 | 1.5658 | 5725.5 | 14.820 | 175.26 | 0.17524 | 0.92604 | 3.24799E−07 | 5.387E−11 | 6029.4 | 971.15 | 0.16107 |
| 0.058376 | 618.34 | 1.6792 | 5939.7 | 24.932 | 165.00 | 0.29481 | 0.87186 | 3.9837E−07 | 6.136E−11 | 6492.2 | 1191.13 | 0.18347 |
| 0.072785 | 808.46 | 2.6364 | 3906.5 | 29.547 | 160.08 | 0.34938 | 0.84582 | 4.36658E−07 | 9.930E−11 | 4397.1 | 1305.61 | 0.29692 |
| 0.086948 | 985.64 | 2.6188 | 3952.3 | 33.617 | 155.57 | 0.39751 | 0.82201 | 4.65932E−07 | 1.015E−10 | 4590.6 | 1393.14 | 0.30348 |
| 0.10036 | 1145.82 | 2.4113 | 4259.6 | 37.091 | 151.59 | 0.43859 | 0.80098 | 4.89519E−07 | 9.591E−11 | 5103.9 | 1463.66 | 0.28677 |
| 0.13427 | 1521.8 | 3.2007 | 3065.6 | 44.473 | 142.61 | 0.52588 | 0.75355 | 5.39745E−07 | 1.353E−10 | 3988.6 | 1613.84 | 0.40461 |
| 0.153925 | 1707.02 | 3.1691 | 2960.8 | 47.983 | 138.04 | 0.56739 | 0.72937 | 5.60209E−07 | 1.384E−10 | 4046.9 | 1675.03 | 0.41390 |

*Values provided by W. Yave for Figure 2 in Yave et al.^8^

# **References for Supporting Information**

1. Gmehling J, Onken U and Rarey-Nies JR, Vapor liquid equilibrium data collection. Aqueous systems (Supplement 2), ed by Behrens D and Eckermann R. DECHEMA, Chemistry Data Series. Vol 1, Part 1b (1988).

2. "DIPPR: Design Institute for Physical Properties." Retrieved 06/10/2022, from <https://www.aiche.org/dippr>.

3. Towler G and Sinnott RK, Appendix C: Physical Property Data Bank, in Chemical Engineering Design - Principles, Practice and Economics of Plant and Process Design (2nd Edition). Elsevier (2013).

4. "Dortmund Data Bank." from <http://www.ddbst.com>. (2017, 05/04/2017).

5. "NIST Chemistry WebBook." Retrieved 06/10/2022, from <https://webbook.nist.gov/chemistry/>.

6. Yaws CL, Yaws' Critical Property Data for Chemical Engineers and Chemists. Online version available at: <https://app.knovel.com/hotlink/toc/id:kpYCPDCECD/yaws-critical-property/yaws-critical-property>. Knovel (2012: 2013: 2014).

7. Kondo M, Komori M, Kita H and Okamoto K, Tubular-type pervaporation module with zeolite NaA membrane. *J Membr Sci* **133**: 133-141 (1997).

8. Yave W, Separation performance of improved PERVAP membrane and its dependence on operating conditions. *Journal of Membrane Science and Research* **5**: 216-221 (2019).

9. DeltaMem-AG, Membrane data sheet: PERVAP^TM^ polymeric membranes (2016).
